# Usefulness of the ^1^H NMR Multisuppression Approach for the Global Characterization of Monovarietal Extra-Virgin Olive Oils

**DOI:** 10.3390/foods13142298

**Published:** 2024-07-22

**Authors:** Encarnacion Goicoechea-Oses, Ainhoa Ruiz-Aracama

**Affiliations:** Food Technology Area, Faculty of Pharmacy, Lascaray Research Center, University of the Basque Country (UPV/EHU), Paseo de la Universidad nº 7, 01006 Vitoria, Spain; encarnacion.goicoechea@ehu.eus

**Keywords:** characterization, extra-virgin olive oil (EVOO), olive cultivar, proton nuclear magnetic resonance (^1^H NMR), main and minor components, phenolic compounds, sterols

## Abstract

Extra-virgin olive oil (EVOO) is one of the most appreciated vegetable oils worldwide, but its high price makes it prone to suffer adulteration with lower quality oils. Therefore, it is important to have methodologies able to study EVOO composition as a whole in a simple and fast way, in order to guarantee its quality and safety. For this purpose, in this study, commercial samples of five Spanish olive cultivars (Arbequina, Arroniz, Cornicabra, Hojiblanca, Picual) were studied by Proton Nuclear Magnetic Resonance (^1^H NMR) spectroscopy, using standard and multisuppression pulses. The aim was to explore the possibility of ^1^H NMR use to characterize in a single run and in a global way the composition of these monocultivar oils, regarding not only their main components (fatty acids supported on triglycerides) but also minor ones (squalene, sterols, diterpenic wax esters of phytol and geranylgeraniol, phenolic and secoiridoid derivatives, like tyrosol, hydroxytyrosol, oleacein, oleocanthal, and lignans, among others, and aldehydes). The use of univariate and multivariate statistical analyses confirmed the presence of compositional features that were specific to some olive varieties. The Arbequina and Arroniz oils showed the most characteristic features that allowed for clearly differentiating them from the others. In contrast, the discrimination between the Cornicabra, Hojiblanca and Picual oils was not so easily achieved.

## 1. Introduction

Extra-virgin olive oil (EVOO) is one of the most appreciated and consumed vegetable oils worldwide, due to the nutritional and sensory properties attributed to it. This traditional Mediterranean food product can be considered a “natural fruit juice”, because it is obtained directly from olive fruits (Olea europaea L.), only by mechanical procedures. EVOO is mainly composed of triglycerides (TG, ~98%), with monounsaturated oleic acid being the main fatty acid (C18:1ꞷ9), and other minor components (~2%), among which sterols, tocols, squalene and phenols, among others [1]. In the last decade, special attention has been paid to the latter phenolic components, because they are considered responsible for some of the health properties attributed to EVOO consumption. In fact, in 2012, the health claim related to olive oil polyphenols regarding their protective effect on blood lipids from oxidative stress was accepted by the European Union (European Commission Regulation No 432/2012, 16 May 2012). According to this health claim, the beneficial effect is obtained with a daily intake of 20 g of olive oil containing at least 5 mg of hydroxytyrosol and its derivatives (e.g., the oleuropein complex and tyrosol; 250 mg/kg oil). Since then, the analysis of these polar phenolic components has become a very challenging area of research, and different protocols have been developed for this purpose, most of them involving previous extraction and separation steps [2]. It should be highlighted that in EVOO of different geographical origins and cultivars, these compounds were detected in very variable concentrations, which appeared affected by climate, the processing of the oil and the storage time and conditions [3].

Considering the above-mentioned properties, it is not surprising that EVOO is a highly demanded oil by consumers worldwide. Regarding olive oil prices, it is remarkable that they have jumped over the last years due to the extreme environmental conditions in the top olive oil-producing countries, which have hampered oil production (the main producer is Spain, followed by Italy and Greece). This quantitative expansion of olive oil consumption and its high price make this oil prone to suffering adulteration with lower quality oils and mislabeling practices [4,5]. Such fraudulent practices can compromise the quality and safety of oil, as well as have significant economic implications. To avoid these practices and guarantee the quality and safety of olive oil, several official standard methods have been implemented, making olive oil one of the most strictly regulated food products [6]. However, in many cases, it is necessary to use different methodologies to characterize and determine the concentration of each type of EVOO components, not only for their extraction and isolation, but also for their separation, identification and quantification. Therefore, in addition to the official methods, it is of paramount importance to explore simple, rapid and environmentally friendly techniques, able to provide qualitative and quantitative information on olive oil main and minor components.

Proton Nuclear Magnetic Resonance spectroscopy (^1^H NMR) has been successfully applied in the last thirty years for this purpose, providing all this information in a single run, without chemical extraction, derivatization or any other modification of the sample [7,8,9]. It must be noted that the main limitation of ^1^H NMR standard experiments is its limited sensitivity when studying minor components that are present in very low concentrations, either naturally present in fresh oil or generated during degradative processes. A way of overcoming this limitation is using multi-signal suppression pulses, which involve the suppression in a selective way of the main spectral signals, which results in the enhancement of the dynamic range [10]. Using a multisupression approach, Ruiz-Aracama et al. [11] carried out a thorough description of the proton signals related to EVOO main (acyl groups) and minor components (squalene, sterols, triterpene acids/esters, fatty alcohols, diterpenic wax esters and phenols such as lignans, tyrosol, hydroxytyrosol, oleocanthal, oleacein, oleokoronal, oleomissional, ligstrodials and oleuropeindials) and also of the proton signals related to compounds generated in hydrolysis and oxidation reactions. Multisupression experiments have also been successfully applied to characterize different monocultivar EVOO from Italy [12] and, more recently, also from Portugal [13] and Greece [14].

In this context, this study addresses the global characterization of five Spanish monovarietal commercial EVOO (Arbequina, Arroniz, Cornicabra, Hojiblanca, Picual), by means of ^1^H NMR standard and multisuppression experiments. All the information obtained about the main and minor components, processed by univariate and multivariate statistical analyses, was used to identify differences regarding the composition of the above-mentioned Spanish oil varieties. This valuable information could be obtained in one run and without any previous modification of the samples and it will be useful for further research on EVOO quality, authentication and fraud detection.

## 2. Materials and Methods

### 2.1. Samples

Thirty-two samples of EVOO of Spanish origin and of different commercial brands were acquired in local supermarkets. Their labels indicated that they were monovarietal and corresponded to five different olive cultivars: Arbequina (*n* = 7, ABQ1-ABQ7); Arroniz (*n* = 6, ARZ1-ARZ6); Cornicabra (*n* = 5, COR1-COR5); Hojiblanca (*n* = 7, HOJ1-HOJ7); and Picual (*n* = 7, PIC1-PIC7).2.2. Proton Nuclear Magnetic Resonance (^1^H NMR) Spectra Acquisition and Study.

### 2.2. Proton Nuclear Magnetic Resonance (1H NMR) spectra acquisition and study

#### 2.2.1. General

^1^H NMR spectra were acquired with a Bruker Avance 400 spectrometer which operated at 400 MHz (Bruker Biospin, Billerica MA, USA). In total, 175 µL of oil was mixed in a 5 mm diameter tube with 425 µL of deuterated chloroform (CDCl_3_) containing tetramethylsylane (0.03%), which was used as a reference compound (Eurisotop, Saint-Aubin, France). CDCl_3_ was used as a solvent because, in contrast to other ones usually employed (water, methanol), it does not react with the phenolic compounds subject of study [8,15]. Standard and multisuppresion ^1^H NMR spectra were acquired for each sample in duplicate and were processed using MestreNova (Mestrelab Research, Santiago de Compostela, Spain).

#### 2.2.2. Standard Experiments (S)

The acquisition parameters of the Free Induction Decay (FID; RD—p(90º)) were the following: dummy scans (DS) = 4; number of scans (NS) = 64; relaxation delay (D1) = 3 s; spectral width (SW) = 6410 Hz; excitation pulse 90º (P1) = 10.75 μs; acquisition time (AQ) = 4.8190 s; and receiver gain (RG) = 14.3. The total acquisition time (AT) was 8 min 38 s. To obtain quantitative results in the shortest possible period of time, in the acquisition of the ^1^H NMR spectra, a very broad range of recycling times and relaxation delays were tested.

#### 2.2.3. Multisuppression Experiments (MS)

The NOESYGPPS experiments were set as previously described [10]. In short, they involved a 1D ^1^H NMR pulse sequence with the suppression of the main spectral signals. The parameters SW, DS and P1 were set as in the standard experiments. However, the AQ and NS were modified to 2.5560 s and 512, respectively, in such a way that the total AT was 1 h 13 min. As in a previous study, an amplitude- and phase-modulated shaped pulse was applied during RD with a frequency spectrum of 38 highly selective bands, each in the 3 Hz RFfield, to achieve the highly selective suppression of multiple dominating lipid signals, leaving the rest of the spectrum undistorted at 0.2 ppm on each side of the suppressed signals. Thus, in comparison to the standard experiments, it was possible to increment the signal-to-noise ratio by about 10 times because the receiver gain increased to RG = 161. In order to be able to compare the intensity of the signals between the S and the MS spectra of each sample, the signal due to bis-allylic protons (G) was not suppressed [7,8,11,15,16,17,18,19,20,21,22,23,24,25,26,27,28,29,30,31,32,33,34,35].

#### 2.2.4. Determination by ^1^H NMR of the Molar Percentage of the Main Acyl Groups and of the Concentration of Some Minor Components

Due to the fact that the area of the ^1^H NMR signal is proportional to the number of protons that generates it; the proportionality constant is the same for all types of hydrogen atoms; and free fatty acids are present in EVOO in very small concentrations, from the standard spectra (S), it was possible to determine the molar percentages of the different kinds of acyl groups in relation to the total number of acyl groups, using as a reference the glyceryl H signal, as described in the Supplementary Materials [7].

**Figure 1:**
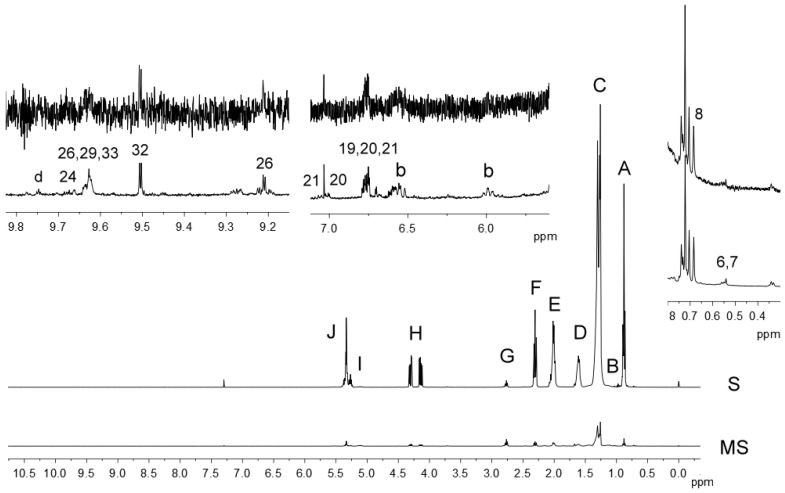
Standard (S) and multisuppressed (MS) ^1^H NMR spectra between 0 and 11 ppm of the sample ARZ5, together with three expanded regions. Using the unsuppressed signal of bis-allylic protons (G) as an internal reference, both spectra were properly scaled. Signal numbers and letters are in agreement with those in Table 1.

Moreover, using the ^1^H NMR spectral data, the concentration of the minor components present in the EVOO could also be determined from the MS spectra. However, to quantify these components, the signal of the glyceryl backbone protons (H signal) could not be used as an internal standard because it was one of the suppressed signals. On the other hand, the G signal of bis-allylic protons was not suppressed; so, its intensity remained quantitative and proportional to the number of protons generating it, as in the standard ^1^H NMR spectrum, where this signal was small but perfectly observable (see Figure 1). Therefore, the intensities of the compounds of interest in the MS ^1^H NMR experiments could be indirectly related to those in the quantitative S experiments, integrating the signal of bis-allylic protons (G) in both spectra of each sample. In this work, the concentration of several EVOO minor components was determined as indicated in the Supplementary Materials and is given in μmol of compound/mol of TG [36]. For those signals due to phenolics (6.59–6.63, 6.76–6.80 and 7.00–7.03 ppm), for which the number of protons generating them was unknown, the concentration was estimated as if only one proton was generating them.

### 2.3. Statistical Analysis

Analysis of variance (ANOVA) was performed using the statistics package IBM SPSS Statistics Version 28.0.1.1 (SPSS Inc., Chicago, IL, USA). Post hoc testing (*p* < 0.05) of the multiple comparisons was performed by Tukey’s B test. An unsupervised multivariate Principal Component Analysis (PCA) was performed using SIMCA P+ version 17 (Sartorius, Goettingen, Germany).

Some inclusion and exclusion criteria were considered for the statistical analysis. The EVOO samples were commercially purchased, being all samples within the time limit of preferential consumption indicated in the label. However, the occurrence of oxidation or degradation processes in some of them, regardless of their varietal origin, could not be excluded. To be able to identify compositional features in the different monovarietal EVOO studied, the samples showing a significant degradation had to be excluded from the statistical analysis in order to avoid bias in the results. Thus, all samples were examined to detect signals related to degradation, and HOJ1, HOJ4 and PIC4 were discarded, as explained below. Only those samples without evident signals of degradation and/or oxidation were included in the statistical study (*n* = 29).

## 3. Results and Discussion

Several authors have related the EVOO composition, regarding both major and minor components, to the olive cultivar. So, the aim of this study was to characterize five Spanish single-cultivar EVOO and to evaluate if a relationship between the content of some oil components, determined by ^1^H NMR spectroscopy, and the olive variety could be found, in order to determine the possibilities offered by this technique. For this purpose, the S and MS ^1^H NMR spectra of 32 monovarietal EVOO samples of five different varieties were acquired; the signals observed in the spectra were assigned, and the corresponding compounds generating them were quantified. As an example, Figure 1 shows both the S and the MS spectra of the ARZ5 sample.

In the S spectra of the EVOO samples, signals due to the main components, i.e., acyl groups esterified with glycerol, as well as to some minor components, such as squalene, 1,2-diglycerides and β-sitosterol, were observed and quantified. However, it is in the MS spectra of EVOO that signals due to several minor components, many of which were not observable in the S spectra, became visible [11,29,33]; therefore, their concentration could be determined from the MS spectra. Table 1 shows the assignment of some chemical shifts and multiplicities of the ^1^H NMR signals, with those used for quantification purposes highlighted in bold; Figures S1 and S2 display the chemical structures of the compounds responsible for those signals.

**Table 1:** Assignment of the chemical shifts, multiplicities and coupling constant (J) of the ^1^H NMR signals (CDCl_3_) of some compounds present in EVOO or generated during processing and storage, using standard compounds or literature data [7,8,11,15,16,17,18,19,20,21,22,23,24,25,26,27,28,29,30,31,32,33,34,35]. The letters and numbers of the signals agree with those in Figure 1 and Figure 2. Supplementary Figures S1 and S2 show some chemical structures together with their carbon atom numbers. The chemical shifts in bold are those used for quantification purposes.

Signal	Compound	Chemical Shift (ppm)	Multiplicity (J in Hz)	Functional Group (Carbon Atom)
Triacylglycerides (TG)				
**A**	Saturated, oleic and/or ω-7 AG	0.879	t	-C**H**_3_
**A**	Linoleic AG	0.889	t	-C**H**_3_
**B**	Linolenic AG	0.972	t	-C**H**_3_
**C**	AG	1.221–1.419	m *	-(C**H**_2_)_n_-
**D**	AG	1.522–1.700	m	-OCO-CH_2_-C**H**_2_-
**E**	AG	**1.941–2.139**	m **	-C**H**_2_-CH=CH-
**F**	AG	**2.305**	dt	-OCO-C**H**_2_-
**G**	G1: Linoleic AG G2: Linolenic AG	**2.765** **2.801**	t t	=HC-C**H**_2_-CH=
**H**	Glyceryl group of TG	4.139, 4.303	dd, dd	-C**H**_2_-OCO-R (C1, C3)
**I**	Glyceryl group of TG	5.270	m	>CH-OCO-R (C2)
**J**	AG	5.296–5.470	m	-C**H**=C**H**-
Sterols and other terpenic compounds				
**1**	Cycloeucalenol ^§^	0.150 **0.390** 3.210 4.660, 4.720	d d dd s, s	-C**H**_2_- (C19) -C**H**_2_- (C19) >C**H**-OH (C3) >C=C**H**_2_ (C28)
**2**	Cycloartenol ^†^	**0.334** 0.555 3.270	d d m	-C**H**_2_- (*exo*, C19) -C**H**_2_- (*endo*, C19) >C**H**-OH (C3)
**3**	24-Methylene-cycloartanol ^§^	**0.334** 0.555 3.270 4.660, 4.720	d d m s, s	-C**H**_2_- (*exo*, C19) -C**H**_2_- (*endo*, C19) >C**H**-OH (C3) >C=C**H**_2_ (C28)
**4**	Cyclobranol ^§^	**0.334** 0.555	d d	-C**H**_2_- (*exo*, C19) -C**H**_2_- (*endo*, C19)
**5**	Esters of cycloartenol ^†^, 24-methylenecycloartanol ^§ ^and cyclobranol ^§^	**0.345** 0.575	d d	-C**H**_2_- -CH_2_- (Cyclopropane ring)?
**6**	Gramisterol ^§^	**0.540** 3.110 4.660, 4.720	s dt s, s	-C**H**_3_ (C18) >C**H**-OH (C3) >C=C**H**_2_ (C28)
**7**	Citrostadienol ^§^, ∆7-Avenasterol ^§^, ∆7-Campesterol ^§^	**0.540** 3.590	s m	-C**H**_3_ (C18) >C**H**-OH (C3)
**8**	β-Sitosterol ^†^, ∆5-Avenasterol ^†^, ∆5-Campesterol ^†^	**0.684** 3.450–3.550	s m	-C**H**_3_ (C18) >C**H**-OH (C3)
**9**	Squalene ^†^	1.670 **5.120**	s m	-C**H**_3_ (C1,C24) >C=C**H**- (C3, C7, C11, C14, C18, C22)
**10**	Obtusifoliol ^§^	**3.110** 4.660, 4.720	ddd s, s	>C**H**-OH (C3) >C=C**H**_2_ (C28)
**11**	Non-cyclic diterpenic esters ^§^	**4.580**	d	=CH-C**H**_2_-OCO- (C1)
Phenolic compounds				
**19**	Hydroxytyrosol ^†^ and derivatives	**6.600** **6.750** **6.780**	dd d (J = 2.0) d (J = 8.0)	-C**H**=
**20**	Tyrosol derivatives ^§^	**6.600** **6.780** **7.015**	d (J = 8.5) d (J = 8.5) d (J = 8.5)	-C**H**=
**21**	Tyrosol ^†^	**6.780** **7.060**	d (J = 8.5) d (J = 8.5)	-C**H**= (C7) -C**H**= (C4)
**22**	Pinoresinol ^†^	6.816 **6.877** 6.895	?	Aromatic protons
**23**	1-Acetoxypinoresinol ^§^	**6.846** 6.884	?	Aromatic protons
**24**	5S,4R-oleuropeindial ^§Ψ^	9.190–9.205 9.670	os d (J = 2.7)	-C**H**O (C1) -C**H**O (C3)
**25**	5S,4S-oleuropeindial ^§Ψ^	9.190–9.205 **9.448**	os d (J = 2.7)	-C**H**O (C1) -C**H**O (C3)
**26**	Oleacein (3,4-DHPEA-EDA) ^§^	**9.209** 9.615–9.645	d (J = 2.0) os	-C**H**O (C1) -C**H**O (C3)
**27**	5S,4R-ligstrodial ^§Ψ^	9.207–9.222 9.680	os d (J = 2.7)	-C**H**O (C1) -C**H**O (C3)
**28**	5S,4S-ligstrodial ^§Ψ^	9.207–9.222 **9.452**	os d (J = 2.7)	-C**H**O (C1) -C**H**O (C3)
**29**	Oleocanthal (p-HPEA-EDA) ^§^	**9.223** 9.615–9.645	d (J = 2.0) os	-C**H**O (C1) -C**H**O (C3)
**30**	S-*E*-Elenolide	**9.270**	d (J = 0.9)	-C**H**O (C1)
**31**	*p*-HPEA-EA ^§^	**9.499**	d (J = 1.8)	-C**H**O (C1)
**32**	3,4-DHPEA-EA ^§^	**9.504**	d (J = 1.8)	-C**H**O (C1)
**33**	Elenolic acid ^§^	**9.615–9.645**	os	-C**H**O (C1)
**34**	Oleomissional ^§Ψ^	7.360 9.190–9.205 **11.780**	dd os d (J = 12.9)	=C**H**-OH (C3) -C**H**O (C1) =CH-O**H** (C3)
**35**	Oleokoronal ^§Ψ^	7.386 9.207–9.222 **11.764**	dd os d (J = 12.9)	=C**H**-OH (C3) -C**H**O (C1) =CH-O**H** (C3)
Compounds derived from oxidative or hydrolytic processes				
**a**	1,2-Diglycerides (glyceryl backbone) ^†^	**3.725**	d/t ***	-C**H**_2_-OH (C3)
**b**	(*Z*,*E*)-conjugated dienes supported in chains having also a hydroperoxide group (OOH) ^†^	5.510 5.560 6.000 6.580	dtm ddm ddtd dddd	-C**H**=C**H**-C**H**=C**H**-
**c**	(*E*)-2-alkenals ^†^	**9.502**	d (J = 7.8)	-C**H**O (C1)
**d**	Alkanals ^†^	**9.750**	t	-C**H**O (C1)
Other signals due to protons in EVOO minor components				
**U**	Unidentified	9.160	s	-C**H**O
**U**	Unidentified	9.266	d/m?	-C**H**O
**U**	Unidentified	9.286	d (J = 1.8)	-C**H**O
**U**	Unidentified	9.310	d (J = 2.0)	-C**H**O
**U**	Unidentified	9.355	d (J = 1.8)	-C**H**O
**U**	Unidentified	9.663	bs/m?	-C**H**O
**U**	Unidentified	9.775	bs?	-C**H**O

Abbreviations: AG: acyl group; bs: broad signal; d: doublet; m: multiplet; os: overlapping signal; q: quartet; s: singlet; t: triplet; 3,4-DHPEA-EDA: dialdehydic form of decarboxymethyl elenolic acid linked to hydroxytyrosol (3,4-dihydroxyphenylethanol); 3,4-DHPEA-EA: oleuropein aglycone 3,4-dihydroxyphenylethanol-elenolic acid; p-HPEA-EDA: dialdehydic form of decarboxymethyl elenolic acid linked to tyrosol (4-hydroxyphenylethanol); p-HPEA-EA: ligstroside aglycone 4-hydroxyphenylethanol-elenolic acid. * Overlapping multiplets of methylenic protons in different acyl chains, either in β-position or, further, in relation to an unsaturation or in γ-position or, further, in relation to the -C=O group. ** Overlapping multiplets of α-methylenic protons in relation to a single double bond of unsaturated acyl chains. *** Standard 1,2-dioleoin and 1,2-dipalmitin give a triplet, whereas in oil samples, this signal can be a doublet or a triplet. ^Ψ^, See reference [18] for more detailed chemical shift assignment. ^†^ Standard compounds were acquired commercially and their ^1^H NMR spectra are shown in the Supplementary Materials. ^§^, Tentatively assigned.

**Figure 2:**
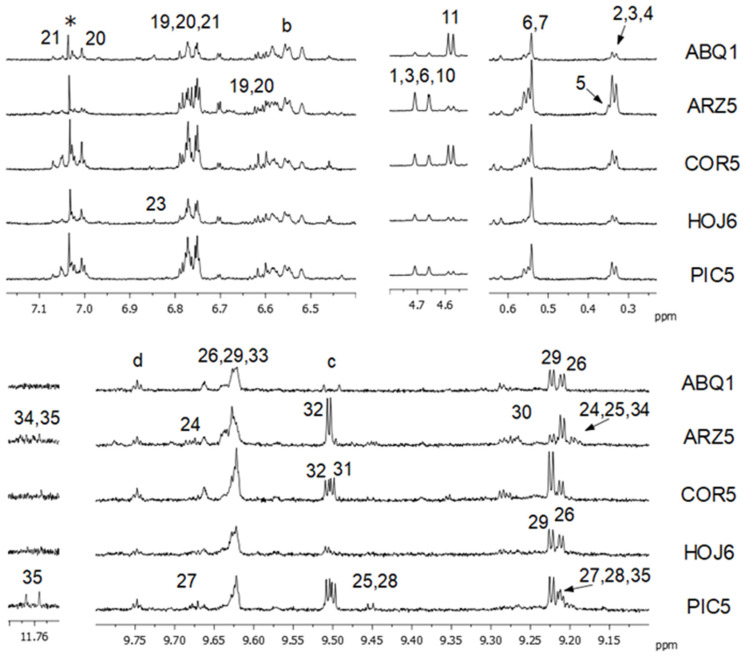
Some ^1^H NMR expanded spectral regions of certain EVOO samples (ABQ1, ARZ5, COR5, HOJ6, PIC5) acquired using the multisuppression approach. See Table 1 for the assignment of signal numbers and letters. * Asterisked peak is a side band of chloroform.

The molar percentage of the main acyl groups, as well as the concentration of several minor components (expressed as μmol of compound/mol of TG) in each EVOO sample are given in Table S1. As can be observed, some compounds, such as sterols and terpenic compounds, were present in all or almost all oil samples. In contrast, other oil components, like phenolic compounds supporting aldehydic groups in their structure, were present in the different samples in more variable concentrations, as shown in Figure 2, which depicts representative MS spectral regions of one EVOO sample for each cultivar. Several authors have suggested that it is possible to discriminate among different monovarietal olive oils, taking into account their content of major and/or minor components. Thus, in this study, in order to check the usefulness of ^1^H NMR to determine whether there is any relationship between the olive cultivar and the oil composition, univariate and multivariate statistical analyses were performed.

### 3.1. Univariate Analysis: Analysis of Variance (ANOVA)

The potential relationship between oil composition and varietal origin was investigated by performing an analysis of variance (ANOVA) on the quantitative data for major and minor components, determined from both spectra of the EVOO samples (S and MS). It must be noted that before carrying out this statistical analysis, the degradative status of the samples was checked, since only those without evident signs of oxidation or degradation could be considered as representative of each variety and, therefore, included in the ANOVA study.

Regarding lipid oxidation, during oil storage, several compounds of different nature can form and, if present in a high enough concentration, will give proton signals visible in the ^1^H NMR spectra. Well-known signals produced by oxidation compounds are those of conjugated dienes supported on chains containing also a hydroperoxide group, which are called primary oxidation products. These signals were not present in the S spectra, but they were visible in the MS spectra of all the EVOO samples, evidencing the low oxidative status of all samples (see signal b in Figure 1). Anyway, these signals should not be considered a variable for the ANOVA, because the presence of these oxidation compounds is not related to the olive variety and could distort the results of the statistical analysis, as was already pointed out previously [37].

Furthermore, in the MS spectra of some EVOO samples (HOJ1, HOJ4 and PIC4), two unassigned doublets were observed at 9.310 and 9.355 ppm. Their presence coincided with the absence or almost absence of the aldehydic protons signals of the dialdehydic secoiridoids oleocanthal (p-HPEA-EDA) and oleacein (3,4-DHPEA-EDA), as well as with the absence of the signal at 7.015 ppm, related to tyrosol derivatives (see Figure S3). Therefore, it can be postulated that these doublets were due to compounds generated in the degradation of the above-mentioned phenolic derivatives, as was already suggested by Christophoridou et al. [38]. So, although these three samples did not show a high oxidative status, they were excluded from the statistical analysis in order to obtain sound results regarding the natural phenolic content of each EVOO cultivar. On this basis, an ANOVA (*p* < 0.05) was performed on the remaining samples (*n* = 29). The results are shown in Table 2, and the most characteristic compositional features of each monocultivar oil are discussed below. It should be noted that out of the 32 variables that were considered for the statistics, 27, i.e., 84% of them, were significantly different amongst the varieties.

#### 3.1.1. Differences in Composition Found for the Arbequina EVOO Samples

As can be observed in Table 2, the Arbequina oil samples were the ones showing the biggest differences regarding the content of major components amongst the five varieties here studied. They showed a significantly lower proportion of oleic (C18:1ω9) acyl groups than the other monovarietal EVOO, whereas the content of linoleic (C18:2ω6) and, mainly, of saturated groups was significantly higher in this variety, in agreement with what described by other authors [39,40].

With respect to their composition in minor components, several differences were found amongst the EVOO samples obtained from the Arbequina and the other four monovarietal oils. In the Arbequina samples, the concentration of all sterols was lower than in the oil samples from the other varieties. As an example, the concentration of 7-sterols (gramisterol, citrostadienol and/or 7-avenasterol) and that of the main 5-sterols (β-sitosterol, 5-avenasterol and/or 5-campesterol) were significantly lower in the Arbequina samples. In addition, differences were also found in relation to the squalene content. Squalene is a triterpenic hydrocarbon with antioxidant properties [41] that is present at high levels in olive oil. Nevertheless, the Arbequina oil showed the lowest squalene content among the five monovarietal oils here studied, which was less than half that shown by the other varieties.

In contrast, the oil from the Arbequina variety showed the highest concentration of diterpenic wax esters (esters of phytol and geranylgeraniol) among the different oils studied, which is in agreement with what reported by other authors [42,43]. Their content was similar to that found in the Cornicabra oil samples and significantly higher than in the other three monovarietal oils (Arroniz, Hojiblanca and Picual). Wax esters are lipids known to be present in the epicarp of the olive fruit and can be transferred to the oil when this is extracted. They are composed of a single fatty acid esterified to a long-chain alcohol; among these latter the most important ones are fatty alcohols (mainly tetracosanol and hexacosanol) and diterpenic alcohols (phytol and geranylgeraniol). It is remarkable that the wax esters of fatty alcohols have traditionally been considered markers of low-quality oils because they are more abundant in oils produced from softer and more degraded olives, being transferred to the oils during mechanical extraction. In addition, and since they are present in higher amount in solvent-extracted olive oils, like pomace oil, they have also been used to detect EVOO adulteration [44]. However, it must be noted that Aragon et al. [45] reported that the Arbequina and Cornicabra oils contain a significantly high concentration of total wax esters of fatty alcohols. This finding, together with the high concentration of diterpenic wax esters found in the present study and in previous ones [42,43], indicate that the content of wax esters strongly depends upon the olive cultivar. Moreover, some authors found no relationship between the profile of wax esters of an oil and that of the corresponding olive epicarp, which refutes the hypothesis that their presence in an oil is solely due to their transfer from the epicarp during mechanical extraction [42]. Thus, wax esters should be considered not only low-quality markers of EVOO, but also markers of certain monocultivar oils.

Regarding phenolics, most signals due to the aromatic protons of hydroxytyrosol and its derivatives and of tyrosol and derivatives overlap [18]; so, they were jointly integrated (see Table 1, Table 2 and Table S1). In the Arbequina oil samples, a lower intensity of the signals of these aromatic protons was observed (see Figure 2). This observation was confirmed by the concentration values of several individual derivatives, such as oleacein (3,4-DHPEA-EDA), oleocanthal (p-HPEA-EDA), the dialdehydic forms of oleuropein and ligstroside aglycones, whose concentrations were found to be lower in the samples of Arbequina EVOO (see Table 2). Regarding oleomissional and oleokoronal, which are monoaldehydic derivatives of hydroxytyrosol and tyrosol, respectively, they were not detected in the Arbequina oil samples. In addition, 3,4-DHPEA-EA (monoaldehydic form of oleuropein aglycone) and p-HPEA-EA (monoaldehydic form of ligstroside aglycone) were detected only in a few samples in very low concentration. Other authors have also reported the absence or almost absence of the signal of p-HPEA-EA in Arbequina monocultivar oils [46,47]. The fact that these oil samples only contained the dialdehydic forms of ligstroside and oleuropein aglycones but not the monoaldehydic forms suggests that the metabolic pathways synthesizing these two types of aldehydes (dialdehydes and monoaldehydes) can be different [15].

On the other hand, other phenolic compounds were found to be present in a significantly higher concentration in the Arbequina oil samples than in the other monovarietal oils. Thus, although poor in phenols derived from tyrosol and hydroxytyrosol, the Arbequina samples showed significantly higher concentrations of the lignan 1-acetoxypinoresinol, another phenolic compound described in olive oil, which is in agreement with what found by other authors [46,47,48].

Finally, also worth mentioning is the content of (E)-2-alkenals, which was also higher in the Arbequina oil samples than in the others. This finding is in agreement with those described by other authors [39,47,49], who found higher concentrations of E-2-hexenal in Arbequina monovarietal oils than in oils obtained from other olive varieties, such as Picual or Cornicabra. Indeed, E-2-hexenal has been associated with green fruit notes that are part of the characteristic sensory descriptors of EVOO from Arbequina olive cultivars [39,50].

#### 3.1.2. Differences in Composition Found for the Arroniz EVOO Samples

The Arroniz olive variety (also known as Hembra, Vidrial or Royuela) is typical of some regions in the north of Spain (south of the Basque Country, Navarra or La Rioja). Although scarcely studied, the number of research articles studying the Arroniz olive oil has increased in the last years, as this oil is resistant to cold and drought [51,52].

Regarding its composition in major components, given in Table 2, the Arroniz oil showed a lower proportion of saturated groups than the other four monovarietal oils here studied, but a higher percentage of linoleic groups than the Cornicabra, Hojiblanca and Picual oils, being only surpassed by the Arbequina EVOO.

As far as the concentration of minor components is concerned, several significant differences were found between the Arroniz EVOO and the oils obtained from the other cultivars. The content of squalene in the Arroniz oil samples was significantly higher than in the other four monovarietal oil samples. This high squalene content found in oils from the Arroniz variety is in agreement with the results described by Beltran and coworkers [51], who classified the Arroniz oil as a “high-squalene-content oil”. However, in that study, no statistically significant differences were found between Arroniz, Picual and a Cornicabra EVOO regarding the concentration of this compound.

In relation to sterols and other terpenic compounds, the Arroniz samples showed the highest content of 4,4-dimethylsterols bearing a 9β,19-cyclopropane structure, such as cycloartenol, 24-methylene-cycloartenol and cyclobranol (see signals 2, 3 and 4 in Figure 2). This is a remarkable fact, since these compounds are related to the biosynthesis of phytosterols, as was already postulated by Goad et al. [53]. Indeed, cycloartenol plays a role in the biosynthesis of sterols in plants, being the first cyclation product of squalene, which is subsequently modified to give different phytosterols. The Arroniz oil samples also showed the highest content of ∆7-sterols, such as gramisterol, citrostanediol, ∆7-campesterol and/or ∆7-avenasterol, also in agreement with their involvement in the squalene pathway [54].

Furthermore, differences regarding phenolic compounds were found when the Arroniz oil samples were compared to those obtained from other cultivars. The significantly higher content of hydroxytyrosol and its derivatives found in these oil samples, determined jointly from their overlapping aromatic signals, is noteworthy. In addition, the concentration of several individual secoiridoid derivatives of hydroxytyrosol, such as oleacein (3,4-DHPEA-EDA), 3,4-DHPEA-EA and oleomissional, was also significantly higher in the Arroniz oil samples than in the other oil samples studied. On the contrary, the Arroniz samples showed a lower content of tyrosol and its derivatives (oleocanthal -p-HPEA-EDA- and p-HPEA-EA) than the Cornicabra, Hojiblanca and Picual oils, with concentrations only higher than in the Arbequina oil samples (which showed the lowest content of phenolics, as described above). This finding is of special relevance because hydroxytyrosol has been described to be a more potent antioxidant than tyrosol, probably due to the influence of its catechol structure [55,56]. Taking this into account, it could be hypothesized that its derivatives would show a similar behavior, being more potent antioxidants than the tyrosol derivatives.

It must be noted that the Arroniz oil samples also showed the highest concentrations, among the five monocultivar oils, of the secoiridoid derivative E-elenolide and its derived product elenolic acid, although only the difference in the concentration of the latter was statistically significant. According to the biosynthetic pathway proposed by Rigakou et al. [29], elenolide derives from oleokoronal and oleomissional. The significantly higher concentration of both oleomissional and elenolic acid found in the Arroniz samples in comparison with those of other varieties, is in line with the above-mentioned pathway. Regarding lignans, the total absence of both pinoresinol and 1-acetoxypinoresinol is remarkable, as these compounds were present in variable amounts in the other four monovarietal oils.

Finally, the content of 1,2-diglycerides estimated in the Arroniz oil samples, although low, was found to be higher than in the other monovarietal oils.

#### 3.1.3. Differences in Composition Found for the Cornicabra EVOO Samples

The Cornicabra oil samples showed the highest content of oleic acyl groups and the lowest content of linoleic amongst all the monovarietal EVOO here studied (see Table 2), which is in agreement with what found by other authors [57].

In relation to minor components, the Cornicabra oil showed a slightly lower content of squalene than the Arroniz oil, which, however, was still high. Indeed, oils obtained from Cornicabra variety were also classified as “high-squalene-content” oils in the study carried out by Beltrán and coworkers [51]. Regarding sterols and terpenic compounds, in this monovarietal oil, as in the above-described ones, the main sterols were −5 sterols, and their content was similar to that in the Arroniz, Hojiblanca and Picual EVOO. Nevertheless, a remarkable feature of the Cornicabra olive oil was the high content in diterpenic wax esters (esters of phytol and/or geranylgeranyol), which was similar to that found in the Arbequina oil, as described above and reported by other authors [42].

With respect to phenolics, the Cornicabra EVOO samples showed the highest content of tyrosol and its derivatives among the five monocultivar oils studied, in agreement with other studies [46,58]. This was reflected in the intensity of the signals due to the phenolic protons of these compounds (at 7.015 ppm for tyrosol and 7.060 ppm for the derivatives of tyrosol), as well as to oleocanthal (p-HPEA-EDA), whose concentration was significantly higher than in the other EVOO (see signal 29 in COR5 in Figure 2). Furthermore, the concentration of p-HPEA-EA was also higher in the Cornicabra oil samples than in the Arbequina, Arroniz and Hojiblanca ones, but it was very similar to that found in Picual oil.

Finally, 1-acetoxypinoresinol and pinoresinol, which are two lignans relatively abundant in the Cornicabra olive oil, were also found in the samples of this variety here studied. However, whereas some authors found that the content of pinoresinol in the Cornicabra EVOO was only slightly higher than that in the Picual or Arbequina oils [58], in this study, the concentration of pinoresinol was found to be three times higher in the Cornicabra samples than in the oil samples of the other two varieties. Furthermore, 1-acetoxypinoresinol was found to be present at a concentration significantly lower in the Cornicabra oil samples than in the Arbequina or Hojiblanca samples, in agreement with what described by Gómez-Alonso et al. [58].

#### 3.1.4. Differences in Composition Found for the Hojiblanca EVOO Samples

As can be observed in Table 2, the Hojiblanca EVOO showed a molar percentage of linolenic groups (C18:3ω3) slightly higher, and significantly, than the other monovarietal olive oils. For the rest of the acyl groups, their content in the Hojiblanca oil samples can be taken as corresponding to the average of those of the other olive oil samples here studied.

Regarding the concentration of minor components, the squalene content was relatively high in the Hojiblanca oil samples, similar to those in the Cornicabra and Picual oils, lower than in the Arroniz oil and significantly higher than in the Arbequina oil. Regarding sterols, Δ5-sterols were found, again, to be the main sterols, as it occurred with the other varieties studied. These, together with Δ7-sterols, were found in similar concentrations as in the Arroniz, Hojiblanca and Picual oils, showing values significantly higher than in the Arbequina oil. In contrast, the concentration of 4,4-dimethylsterols was similar to that in the Cornicabra and Picual oils and higher than in the Arbequina oil, but significantly lower than in the oil from the Arroniz cultivar. In relation to diterpenic wax esters, the Hojiblanca oil samples showed a similar concentration as in the Arroniz and Picual oils, but a significantly lower concentration than in the Arbequina and Cornicabra oils, as indicated above.

The Hojiblanca EVOO showed a relatively high concentration of the phenolics tyrosol, hydroxytyrosol and their derivatives (determined from their phenolic signals). It is worth noting that this oil is the only one, together with the Arroniz oil, containing oleomissional, the monoaldehydic secoiridoid derived from hydrotyrosol (see Table 2 and Table S1). Furthermore, the concentration of the lignan 1-acetoxypinoresinol was found to be significantly higher in the Hoijblanca oil than in the Arroniz, Cornicabra and Picual monocultivar oils; on the contrary, pinoresinol showed a much lower concentration. These results are in line with those reported by other authors who determined the most relevant phenolic compounds in the Hojiblanca, Cornicabra, Arbequina and Picual EVOO [58].

#### 3.1.5. Differences in Composition Found for the Picual EVOO Samples

The composition in acyl groups of the Picual EVOO, as shown in Table 2, was found to be similar to that of the Cornicabra oil, with a high molar percentage of oleic groups and a low percentage of linoleic groups.

In relation to the minor components, the concentration of squalene was, again, much higher than in the Arbequina oil samples, but lower than in the Arroniz, Cornicabra and Hojiblanca olive oils. The main sterols detected were Δ5-sterols, with also a relatively high content of 4,4-dimethylsterols bearing a 9β,19-cyclopropane structure. In general, the Picual monocultivar oil samples showed a similar sterol content as the Cornicabra and Hojiblanca oils, but a sterol higher content than the Arbequina oil samples, in contrast to what reported by Alvarruiz and coworkers [40], who found significantly lower values for total sterols in the Picual oil than in the Arbequina and Cornicabra cultivar oils.

Several authors reported that the Picual oil is rich in phenolic compounds [46,58], which is in agreement with what observed in this work. Indeed, hydroxytyrosol, tyrosol and their derivatives, determined from their joined signal of phenolic protons at 6.78 ppm, showed the highest concentration among the different EVOO here studied (see PIC5 sample in Figure 2). In addition, the Picual oil samples also showed the highest concentrations of monaldehyidic derivatives derived from hydroxytyrosol and tyrosol (3,4-DHPEA-EA and p-HPEA-EA, respectively). It must be noted that the latter compound is responsible for the pungent sensation at the back of the throat produced by certain EVOO [59], and the Picual oil has been described to provoke this sensation, which is very important for consumer acceptation [39].

As for other phenolic compounds, both lignans 1-acetoxypinoresinol and pinoresinol were found to be present in the Picual EVOO samples, although in lower concentrations than in other monovarietal oils here studied.

### 3.2. Multivariate Analysis: Principal Component Analysis (PCA)

**Figure 3:**
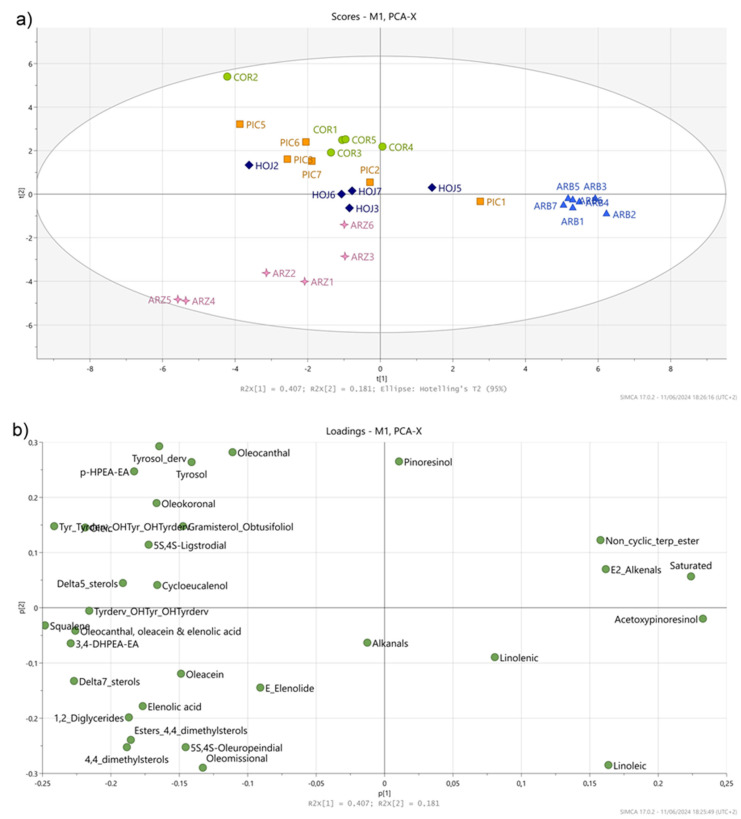
Principal Component Analysis (PCA) (**a**) score and (**b**) loading plots of the first two principal components, performed on the mean abundance values of the main and minor components detected in the EVOO of the Arbequina (ABQ), Arroniz (ARZ), Cornicabra (COR), Hojiblanca (HOJ) and Picual (PIC) varieties.

All the above-described information was obtained from the univariate analysis performed on the quantitative information for each compound, determined from the S and MS spectra of the oil samples. However, multivariate analysis, such as Principal Component Analysis (PCA), is able to analyze several compositional features simultaneously (variables) to identify relationships between them, thus revealing interdependencies. This makes it well suited for detecting interactions that univariate analysis might miss. In addition, it provides a graphical visualization of the similarities and differences between the data and, therefore, facilitates their interpretation. PCA, built with the 28 variables above-mentioned, gave component scores and loadings which are displayed in scatter plots in Figure 3. The score plot of t1/t2 (Figure 3a) shows that there was a good clustering of the Arbequina samples along t1, whereas the Arroniz EVOO samples were well separated from the Cornicabra, Hojiblanca and Picual samples along the second component, t2. On the other hand, the samples of the oils of Cornicabra, Hojiblanca and Picual cultivars almost overlapped in this PCA and could not be separated.

The analysis of the loading plot, containing the underlying loadings responsible for the separation of the samples in this PCA, allowed for the identification of the compounds that contributed to the observed separation of the oil samples (see Figure 3b). The separation of the Arbequina EVOO samples could be attributed to the higher proportions of saturated and linoleic acyl groups, as well as to the higher concentrations of (E)-2-alkenas, 1-acetoxypinoresinol and diterpenic wax esters in comparison to the other oil samples, in agreement with what inferred from Table 2.

The separation of the Arroniz samples could be mainly attributed to 4,4-dimethylsterols and their esters (such as cycloartanol, 24-methylene-cycloartenol and cyclobranol), as well as to Δ7-sterols. As commented above, the contribution of the hydroxytyrosol secoiridoid derivatives (oleomissional, 5S,4S-oleuropeindial and oleacein), as well as that of E-elenolide and elenolic acid, to the separation of the Arroniz samples from all the other oil samples, both in t1 and in t2, is remarkable. On the contrary, the variables related to tyrosol and its derivatives (oleokoronal, 5S,4S-ligstrodial and oleocanthal) contributed positively to the separation of the Cornicabra, Hojiblanca and Picual samples.

As for the other three monocultivar oils (Cornicabra, Hojiblanca, Picual), no separation was observed in the score plot. However, the position of the different samples of these three varieties in the PCA plot also gave some interesting information when combined with the information of Table S1. Thus, and taken as an example, the Picual oil samples from PIC2 to PIC7 were grouped in the lower left-hand side of the plot, whereas the position of the sample PIC1 was closer to that of the cluster of the Arbequina samples. The separation of PIC1 from the rest of the Picual samples could give an indication of a somehow different composition. As can be observed in Table S1, PIC1 contained a higher proportion of linoleic and saturated acyl groups and higher amounts of 1-acetoxypinoresinol and diterpenic wax esters than the average Picual samples; on the contrary, the content of oleic and squalene was lower. So, based on this information, it cannot be excluded that the PIC1 sample was blended with a certain amount of Arbequina oil.

## 4. Conclusions

These results evidence that a great deal of quantitative information can be obtained from ^1^H NMR standard (S) and multisupresion (MS) spectra, which can be very useful to characterize EVOO in a direct, fast and global way, without any previous sample modification. This approach simplifies the methodology and minimizes the potential artifact formation. It must be highlighted that the multisuppresion approach here employed can be very valuable to enhance NMR sensitivity, when potent spectrometers are not available.

Although the number of samples here studied was limited, and the oils were commercially purchased, the information obtained from the ^1^H NMR spectra, combined with the univariate statistical analysis, made it possible to detect compositional features specific of certain cultivars. The results obtained are in agreement with many other previous studies, but it must be noted that in the latter, usually different techniques were employed to study the several types of EVOO components. Furthermore, the PCA plot of the multivariate statistical analysis also showed intra-variety variability of the oil samples or even outlier samples that might correspond to unlabeled blends marketed as single-varietal oils, thus enabling fraud to be detected.

Taking into account all of the above, with a larger sample size, preferably obtained directly from olive oil mills, the methodology described here would make it possible to differentiate between EVOO not only of different varieties, but also of different geographical origins and harvests or obtained by different technological processes.

## Figures and Tables

**Table 2:** Average composition of the main and minor components of EVOO of the Arbequina (ABQ), Arroniz (ARZ), Cornicabra (COR), Hojiblanca (HOJ) and Picual (PIC) varieties, estimated from the ^1^H NMR data. Data regarding the main acyl groups are expressed as molar percentages, and data regarding olive oil minor components are expressed in micromol of compound per mol of triglyceride (TG). Different letters within each row of the five columns indicate a significant difference (*p* < 0.05).

	ARB	ARZ	COR	HOJ	PIC
Molar percentage of main acyl groups					
Linolenic	0.6 ± 0.0 ^a^	0.6 ± 0.1 ^a^	0.5 ± 0.0 ^a^	0.7 ± 0.0 ^b^	0.6 ± 0.1 ^a^
Linoleic	9.3 ± 1.0 ^c^	7.7 ± 0.6 ^c^	3.2 ± 0.4 ^a^	5.9 ± 0.7 ^b^	4.4 ± 2.1 ^a,b^
Oleic	74.6 ± 1.7 ^a^	81.6 ± 1.0 ^b,c^	84.7 ± 1.0 ^b^	81.2 ± 1.9 ^b^	83.2 ± 3.5 ^b,c^
Saturated	15.4 ± 0.9 ^c^	10.0 ± 0.7 ^a^	11.5 ± 0.6 ^a,b^	12.2 ± 1.6 ^b^	11.9 ± 1.4 ^b^
µmol of sterols and other terpenic compounds/ mol TG					
Cycloeucalenol	4.5 ± 3.1 ^a^	18.7 ± 11.7 ^a,b^	26.0 ± 8.6 ^b^	20.6 ±9.5 ^a,b^	19.4 ± 13.9 ^a,b^
4,4-Dimethylsterols *	161.9 ± 53.9 ^a^	758.4 ± 190.6 ^c^	300.3 ± 113.0 ^a,b^	267.1 ± 113.9 ^a,b^	375.9 ± 93.4 ^b^
Esters of 4,4-dimethylsterols *	44.3 ± 11.0 ^a^	226.6 ± 63.9 ^b^	105.3 ± 49.8 ^a^	100.0 ± 36.5 ^a^	112.4 ± 34.4 ^a^
Gramisterol + Obtusifoliol	117.1 ± 41.9 ^a^	172.1 ± 81.3 ^a^	268.5 ± 33.3 ^b^	329.0 ± 75.8 ^b^	282.6 ± 74.4 ^b^
∆7-Sterols *	85.9 ± 13.8 ^a^	193.8 ± 30.9 ^b^	147.5 ± 32.8 ^b^	164.9± 27.2 ^b^	148.1 ± 32.0 ^b^
∆5-Sterols *	1123.7 ± 108.2 ^a^	1521.8 ± 80.4 ^b^	1599.6 ± 157.3 ^b^	1619.0 ± 38.3 ^b^	1460.1 ± 118.2 ^b^
Squalene	7342.9 ± 919.3 ^a^	18,755.6 ± 1592.0 ^b^	16,360.0 ± 1345.4 ^b^	16,360.0 ± 2922.9 ^b^	15,455.6 ± 2622.4 ^b^
Non-cyclic diterpenic esters (wax esters)	804.4 ± 87.9 ^b^	351.0 ± 117.2 ^a^	913.2± 154.6 ^b^	409.0 ± 207.7 ^a^	326.7 ± 283.8 ^a^
µmol of phenolic compounds/ mol TG					
Pinoresinol	5.9 ± 5.7 ^a^	0.1 ± 0.2 ^a^	15.8 ± 4.5 ^b^	2.7 ± 6.0 ^a^	5.8 ± 6.8 ^a^
1-Acetoxypinoresinol	30.8 ± 5.7 ^b^	0.0 ± 0.0 ^a^	4.2 ± 5.7 ^a^	19.0 ± 12.9 ^b^	4.6 ± 11.2 ^a^
5S,4S-oleuropeindial	0.0 ± 0.0 ^a^	10.7 ± 8.7 ^b^	0.0 ± 0.0 ^a^	3.5± 7.8 ^a,b^	0.0± 0.0 ^a^
Oleacein (3,4-DHPEA-EDA)	67.2 ± 30.6 ^a^	152.4 ± 79.1 ^a^	80.0 ± 62.7 ^a^	107.4 ± 38.2 ^a^	87.0 ± 19.7 ^a^
5S,4S-ligstrodial	0.0 ± 0.0 ^a^	13.5 ± 10.7 ^a^	22.5 ± 22.1 ^a^	16.2 ± 15.1 ^a^	9.2 ± 17.3 ^a^
Oleocanthal (*p*-HPEA-EDA)	89.1 ± 19.6 ^a^	93.9 ± 57.5 ^a^	192.0 ± 68.6 ^b^	157.1 ± 57.2 ^a,b^	158.7 ± 56.5 ^a,b^
*E*-Elenolide	6.3 ± 8.3 ^a^	73.5 ± 94.7 ^a^	23.4 ± 27.5 ^a^	53.5 ± 35.8 ^a^	8.3 ± 14.4 ^a^
*p*-HPEA-EA	3.1 ± 8.1 ^a^	33.5 ± 17.4 ^a,b^	79.6 ± 64.1 ^b^	42.8 ± 27.1 ^a,b^	87.8 ± 42.7 ^b^
3,4-DHPEA-EA	4.9 ± 9.0 ^a^	165.6 ± 107.5 ^b^	86.0 ± 53.6 ^a,b^	71.0 ± 35.8 ^a,b^	138.7 ± 47.0 ^b^
Oleacein + Oleocanthal + Elenolic acid	200.7 ± 57.1 ^a^	496.3 ± 153.5 ^b^	418.6 ± 129.4 ^b^	344.5 ± 88.9 ^a,b^	331.2 ± 88.3 ^a,b^
Elenolic acid (estimated)	44.4 ± 50.9 ^a^	250.0 ± 106.9 ^b^	146.7 ± 55.3 ^a^	80.0 ± 64.8 ^a^	85.5 ± 34.1 ^a^
Oleokoronal	0.0 ± 0.0 ^a^	25.8 ± 13.3 ^a^	52.8 ± 46.2 ^a^	53.6 ± 29.4 ^a^	48.0 ± 48.0 ^a^
Oleomissional	0.0 ± 0.0 ^a^	46.0 ± 26.5 ^b^	0.0 ± 0.0 ^a^	27.6 ± 18.2 ^b^	0.0 ± 0.0 ^a^
Tyrosol (7.06 ppm)	92.8 ± 66.7 ^a^	139.4 ± 30.8 ^a^	400.6 ± 213.9 ^b^	261.5 ± 80.3 ^a,b^	337.5 ± 143.7 ^b^
Estimated concentration of phenolic compounds (µmol/ mol of TG)					
Tyrosol derivatives (7.015 ppm)	254.2 ± 56.6 ^a^	384.3 ± 122.9 ^a^	855.2 ± 307.4 ^b^	758.1 ± 327.9 ^b^	758.5 ± 277.1 ^b^
Phenolics at 6.78 ppm: Tyr, Tyr derivatives, OHTyr and OHTyr derivatives *	624.3 ± 196.2 ^a^	1610.8 ± 417.8 ^b^	1856.8 ± 634.0 ^b^	1712.7 ± 634.1 ^b^	1977.6 ± 540.5 ^b^
Phenolics at 6.60 ppm: OHTyr, OHTyr derivatives, Tyr derivatives *	331.6 ± 100.4 ^a^	736.0 ± 281.0 ^b^	588.0 ± 205.5 ^a,b^	694.3 ± 248.4 ^b^	688.1 ± 109.6 ^b^
µmol of other minor compounds/ mol TG					
1,2-Diglyerides	5642.0 ± 528.1 ^a^	9053.1 ± 1404.2 ^c^	6149.7 ± 1168.9 ^a^	7913.6± 1035.6 ^b,c^	6888.8 ± 960.7 ^a,b^
(*E*)-2-alkenals	32.11 ± 7.2 ^b^	13.5 ± 3.6 ^a^	19.6 ± 8.6 ^a,b^	18.2 ± 8.1 ^a^	15.9 ± 12.6 ^a^
Alkanals	61.5 ± 17.1 ^a^	72.5 ± 14.8 ^a^	56.5 ± 16.4 ^a^	70.9 ± 8.9 ^a^	81.4 ± 43.7 ^a^

* Abbreviations: 4,4-dimethylsterols: cycloartenol+ 24-methylene cycloartanol + cyclobranol; ∆7-sterols: gramisterol + citrostadienol + ∆7-avenasterol; ∆5-sterols: β-sitosterol + ∆5-avenasterol + ∆5-campesterol; OHTyr: hydroxytyrosol; Tyr: tyrosol.

## Data Availability

The original contributions presented in the study are included in the article/Supplementary Material, further inquiries can be directed to the corresponding author.

## Supplementary Materials

The following supporting information can be downloaded at: https://www.mdpi.com/article/10.3390/foods13142298/s1. Equations for quantification from the ^1^H NMR spectral data; Table S1: Quantitative data obtained by ^1^H NMR for the main and minor components of the monovarietal Arbequina (ABQ), Arroniz (ARZ), Cornicabra (COR), Hojiblanca (HOJ) and Picual (PIC) extra-virgin olive oils; Figure S1: Chemical structures of olive oil sterols; Figure S2: Chemical structures of terpenoids, non-cyclic diterpenyl esters and phenolic and secoiridoid derivatives; Figure S3: Two expanded regions of the HOJ1 and PIC4 samples’ multisuppressed ^1^H NMR spectra; ^1^H NMR spectra of the standards of some EVOO components spiked in the oils: cycloartenol, β-sitosterol, Δ5-campesterol, Δ5-avenasterol, squalene, hydroxytyrosol, tyrosol, pinoresinol, 1,2-dioleoylglycerol, (*E*)-2-hexenal and hexanal.
