# Supplementary material for: Usefulness of the 1H NMR Multisuppression Approach for the Global Characterization of Monovarietal Extra-Virgin Olive Oils
Source: Foods. 2024 Jul 22;13(14):2298. doi: 10.3390/foods13142298 (PMC11276439; doi:10.3390/foods13142298)
Supplement: Supplementary file 1 [file foods-13-02298-s001.zip › foods-3087918-supplementary.pdf]

SUPPLEMENTARY INFORMATION

Usefulness of ^{1}H NMR multisuppression approach for the global characterization of monovarietal Extra-Virgin Olive Oils

Encarnacion Goicoechea-Oses, Ainhoa Ruiz-Aracama*

Food Technology, Faculty of Pharmacy, Lascaray Research Center, University of the Basque Country (UPV/EHU). Paseo de la Universidad nº 7, 01006 Vitoria, Spain, Tel.: 34-945-013084. *E-mail: ainhoa.ruiz@ehu.eus

1. Quantification from ^{1}H NMR spectral data

The molar percentage of linolenic (C18:3ω3), linoleic (C18:2ω6), oleic (C18:1ω9) and saturated acyl groups (AG) in relation to the total number of moles of acyl groups present in olive oils were estimated as follows, from standard ^{1}H NMR spectra:

Linolenic %=100* (A_{G2}/ 3*A_H) [eq.S1]

Linoleic %=100* (2*A_{G1}/ 3*A_H) [eq.S2]

Oleic %=100* ((A_E - 2*A_{G1} - A_{G2}) / 3*A_H) [eq.S3]

Saturated %=100 – Linolenic% - Linoleic% – Oleic% [eq.S4]

where A_E, A_{G1}, A_{G2} and A_H are the areas of signals **E**, **G1**, **G2** and **H** indicated in **Table 1.**

In the multisupression approach, it must be noted that signal G due to bis-allylic protons was not suppressed, to be used for comparative purposes of the intensity of the signals between the standard and the multisupressed spectra Thus, the concentration of the several minor components, expressed as µmol/mol of total acyl groups, was estimated from multisupressed ^{1}H NMR spectra by using the following general equation:

Component (µmol/mol of TG)=1000000 * [(A_X/ n) / (A_H/4)] [eq.S5]

where TG means triglyceride, A_X is the area of the signal selected for the quantification of each component, shown in **Table 1**, and n is the number of protons that generate each signal.

Table S1. Quantitative data obtained by ^{1}H NMR on the main and minor components of monovarietal Arbequina (ABQ), Arroniz (ARZ), Cornicabra (COR), Hojiblanca (HOJ) and Picual (PIC) extra-virgin olive oils.

	Molar percentage (%)				µmol / mol of triglyceride					
	**Linolenic**	**Linoleic**	**Oleic**	**Saturated**	**Cycloeucalenol**	**4,4-dimethylsterols**	**Esters of 4,4-dimethylsterols**	**Gramisterol + Obtusifoliol**	**Δ7-sterols**	**Δ5-sterols**
**ABQ 1**	0.6	8.8	74.7	15.9	4.3	132.3	32.5	146.2	77.3	952.6
**ABQ 2**	0.7	10.5	73.0	15.9	1.2	142.6	41.6	115.6	79.9	1104.8
**ABQ 3**	0.7	9.7	74.5	15.2	-	94.1	40.8	179.3	80.6	1078.4
**ABQ 4**	0.6	9.4	75.5	14.6	8.8	176.6	42.4	138.6	74.0	1085.9
**ABQ 5**	0.6	8.3	75.8	15.3	4.9	128.1	38.2	56.8	85.2	1124.5
**ABQ 6**	0.6	10.6	71.9	16.9	7.4	251.8	47.4	106.0	115.0	1252.5
**ABQ 7**	0.7	8.0	77.1	14.3	4.6	208.0	67.1	77.3	89.2	1267.2
**ARZ 1**	0.5	7.2	82.9	9.4	12.3	556.5	167.8	241.7	181.4	1521.1
**ARZ 2**	0.7	7.9	82.1	9.4	19.6	778.1	223.0	252.2	210.9	1595.4
**ARZ 3**	0.6	7.7	82.4	9.4	20.5	668.4	154.9	60.8	180.7	1439.2
**ARZ 4**	0.6	8.5	80.3	10.5	34.6	1002.9	311.0	188.7	228.5	1409.7
**ARZ 5**	0.5	8.3	80.4	10.8	25.3	962.2	293.0	206.2	216.9	1576.7
**ARZ 6**	0.7	6.9	81.8	10.6	-	582.3	210.0	82.9	144.6	1588.8
**COR 1**	0.6	3.4	83.7	12.3	32.8	389.6	153.7	321.3	179.2	1687.4
**COR 2**	0.5	2.8	85.6	11.1	12.6	183.2	47.9	243.6	105.7	1536.3
**COR 3**	0.6	3.7	83.7	12.0	24.2	423.5	132.6	279.2	180.3	1649.9
**COR 4**	0.6	2.8	85.7	10.9	26.4	183.0	55.1	257.9	125.3	1764.4
**COR 5**	0.6	3.5	84.7	11.3	34.0	322.0	137.3	240.3	147.1	1359.8
**HOJ 1**	0.9	6.0	81.3	11.8	5.6	150.3	72.6	222.0	148.2	1480.1
**HOJ 2**	0.7	5.5	79.6	14.3	20.9	362.2	95.7	400.1	196.5	1589.0
**HOJ 3**	0.7	5.1	82.8	11.5	20.6	374.7	133.0	296.2	141.9	1651.6
**HOJ 4**	0.8	5.8	82.4	11.0	3.3	179.1	98.6	271.0	167.0	1761.7
**HOJ 5**	0.7	7.0	79.0	13.2	33.8	297.5	132.7	211.9	134.1	1568.8
**HOJ 6**	0.8	6.1	83.2	10.0	20.7	190.1	94.4	372.9	187.0	1631.4
**HOJ 7**	0.8	5.7	81.6	12.0	7.0	110.7	44.1	363.9	164.9	1654.2
**PIC 1**	0.7	8.6	76.7	14.0	6.6	248.7	100.2	246.9	130.4	1491.7
**PIC 2**	0.7	4.3	82.4	12.6	25.8	465.7	152.9	238.6	120.0	1476.9
**PIC 3**	0.5	2.8	86.6	10.1	35.3	456.0	146.3	334.3	149.2	1312.6
**PIC 4**	0.6	3.6	85.2	10.7	45.1	361.0	125.6	337.6	137.1	1798.0
**PIC 5**	0.5	4.3	83.1	12.1	30.9	451.2	117.4	174.9	159.4	1323.5
**PIC 6**	0.5	3.0	85.9	10.6	0.1	305.0	60.3	372.3	124.1	1590.6
**PIC 7**	0.6	3.4	84.2	11.7	17.5	328.8	97.2	328.8	205.7	1565.8

Table S1. (continued)

	µmol / mol of triglyceride								
	**Squalene**	**Non-cyclic diterpenic wax esters**	**Pinoresinol**	**1-Acetoxypinoresinol**	**5S,4S-oleuropeindial**	**Oleacein (3,4-DHPEA-EDA)**	**5S,4S-ligstrodial**	**Oleocanthal (*p*-HPEA-EDA)**	***E*-Elenolide**
**ABQ 1**	6666.7	724.7	-	29.6	-	88.7	-	103.0	10.4
**ABQ 2**	7066.7	857.9	8.8	26.0	-	80.6	-	66.5	19.9
**ABQ 3**	6933.3	900.7	8.2	24.8	-	38.7	-	84.9	0.1
**ABQ 4**	6533.3	676.7	10.9	25.5	-	42.0	-	83.2	13.7
**ABQ 5**	8000.0	819.4	-	38.2	-	35.2	-	89.0	0.1
**ABQ 6**	7066.7	897.7	13.4	35.7	-	117.3	-	124.7	0.1
**ABQ 7**	9133.3	753.6	0.1	35.9	-	67.8	-	72.6	0.1
**ARZ 1**	19800.0	275.5	-	-	14.3	75.4	21.5	48.5	261.6
**ARZ 2**	20266.7	301.8	-	-	12.6	125.4	17.0	96.5	11.4
**ARZ 3**	20000.0	314.2	-	-	-	52.8	-	40.4	12.8
**ARZ 4**	17866.7	318.7	-	-	17.7	226.1	19.1	89.8	65.0
**ARZ 5**	18466.7	307.6	0.5	-	19.7	196.7	23.6	87.0	56.2
**ARZ 6**	16133.3	588.2	-	-	-	237.7	-	201.4	33.7
**COR 1**	17400.0	1187.9	17.9	-	-	15.1	20.4	78.1	-
**COR 2**	16333.3	847.0	22.7	-	-	160.0	61.0	257.7	41.3
**COR 3**	17200.0	871.6	13.2	-	-	109.3	12.7	197.6	13.4
**COR 4**	16800.0	833.4	13.5	10.9	-	17.4	7.2	195.8	62.3
**COR 5**	14066.7	826.1	11.9	10.2	-	98.0	10.9	230.8	0.1
**HOJ 1**	17200.0	240.9	-	11.9	-	-	-	-	-
**HOJ 2**	15533.3	411.2	-	-	17.4	157.6	31.2	253.1	49.7
**HOJ 3**	16066.7	430.6	-	12.3	-	126.7	-	132.5	53.5
**HOJ 4**	17800.0	301.0	-	13.8	-	-	-	-	-
**HOJ 5**	12066.7	729.1	13.4	30.8	-	83.7	-	113.1	13.6
**HOJ 6**	19466.7	164.8	-	29.3	-	110.5	26.5	165.2	39.8
**HOJ 7**	18666.7	309.2	-	22.6	-	58.7	23.5	121.5	111.2
**PIC 1**	11000.0	885.6	0.1	27.4	-	73.7	-	143.0	0.1
**PIC 2**	13933.3	290.7	6.6	-	-	68.2	12.0	105.8	0.1
**PIC 3**	15800.0	176.1	-	-	-	66.0	-	156.8	0.1
**PIC 4**	16800.0	613.5	19.8	-	-	-	-	-	0.1
**PIC 5**	17733.3	318.9	13.0	-	-	109.0	43.2	251.2	35.3
**PIC 6**	16333.3	146.5	0.1	0.1	-	101.3	-	193.0	0.2
**PIC 7**	17933.3	142.6	14.8	-	-	103.6	-	102.4	14.3

Table S1. (continued)

	µmol / mol of triglyceride							
	p-HPEA-EA	3,4-DHPEA-EA	Oleacein + Oleocanthal + Elenolic acid	Elenolic acid (estimated)	Oleokoronal	Oleomissional	Tyrosol (7.06 ppm)	Tyrosol derivatives (7.015 ppm)
**ABQ 1**	-	-	275.4	83.6	-	-	76.0	274.2
**ABQ 2**	-	-	151.9	4.9	-	-	36.3	173.1
**ABQ 3**	-	-	169.1	45.5	-	-	70.6	247.3
**ABQ 4**	-	-	150.1	24.9	-	-	60.2	223.5
**ABQ 5**	-	-	263.9	139.7	-	-	234.1	322.2
**ABQ 6**	-	11.3	242.1	0.1	-	-	58.1	325.2
**ABQ 7**	21.4	22.9	152.5	12.1	-	-	114.2	214.1
**ARZ 1**	8.3	55.1	274.9	151.0	25.2	75.3	134.2	311.4
**ARZ 2**	43.5	112.6	534.5	312.6	30.7	47.6	122.3	389.0
**ARZ 3**	21.2	120.7	347.7	254.4	29.7	41.1	120.5	240.9
**ARZ 4**	56.8	321.9	643.3	327.3	31.3	44.0	123.0	346.6
**ARZ 5**	41.3	278.1	646.1	362.4	37.9	68.4	200.8	416.6
**ARZ 6**	29.8	104.9	531.6	92.5	-	-	135.8	601.4
**COR 1**	39.9	50.9	292.6	199.4	68.3	-	706.1	541.0
**COR 2**	186.5	169.9	628.5	210.7	123.0	-	520.1	1339.6
**COR 3**	48.3	91.1	394.0	87.1	39.0	-	222.8	874.9
**COR 4**	31.4	29.4	340.2	127.0	33.6	-	200.8	645.9
**COR 5**	91.7	88.8	437.9	109.2	-	-	353.1	874.5
**HOJ 1**	48.3	-	197.4	197.4	14.3	-	221.6	-
**HOJ 2**	88.8	129.2	426.1	15.5	79.1	37.3	396.6	1288.6
**HOJ 3**	43.0	69.8	337.5	78.2	34.4	46.8	207.1	455.3
**HOJ 4**	-	39.0	164.7	164.7	21.1	-	321.9	214.4
**HOJ 5**	32.8	71.8	212.7	16.0	48.8	19.6	198.9	561.6
**HOJ 6**	18.8	47.1	427.4	151.7	18.1	-	236.5	648.0
**HOJ 7**	30.8	36.9	318.6	138.5	87.5	34.2	268.5	837.2
**PIC 1**	30.4	65.0	278.2	61.6	-	-	183.7	474.7
**PIC 2**	70.8	122.8	240.3	66.2	23.5	-	280.4	526.4
**PIC 3**	103.8	130.3	352.5	129.7	-	-	541.9	803.1
**PIC 4**	33.5	42.8	114.2	114.2	-	-	518.8	64.4
**PIC 5**	157.2	199.6	449.2	88.9	86.9	-	451.2	1227.2
**PIC 6**	96.5	178.1	415.7	121.4	116.1	-	194.9	877.7
**PIC 7**	68.3	136.3	251.2	45.2	61.4	-	373.1	642.0

Table S1. (continued)

	Estimated concentration (µmol /mol of triglyceride)			µmol / mol of triglyceride		
	**Tyrosol derivatives (7.015 ppm)**	**Phenolics at 6.78 ppm: Tyr, Tyr derivatives, OHTyr and OHTyr derivatives**	**Phenolics at 6.60 ppm: Tyr derivatives, OHTyr and OHTyr derivatives**	**1,2-diglyerides**	***(E)*-2-alkenals**	**Alkanals**
**ABQ 1**	274.2	636.5	291.1	5806.8	24.2	48.4
**ABQ 2**	173.1	432.9	299.9	6609.0	40.9	63.7
**ABQ 3**	247.3	514.2	264.9	5054.9	28.5	66.4
**ABQ 4**	223.5	471.6	265.3	5923.7	26.4	66.1
**ABQ 5**	322.2	1018.1	333.8	5442.0	38.6	61.5
**ABQ 6**	325.2	684.0	552.2	5487.7	39.5	90.0
**ABQ 7**	214.1	612.3	314.1	5169.8	26.7	34.6
**ARZ 1**	311.4	1185.3	466.6	10348.4	17.1	51.6
**ARZ 2**	389.0	1270.2	492.8	9669.7	15.7	77.3
**ARZ 3**	240.9	1470.1	492.0	7890.2	12.0	61.2
**ARZ 4**	346.6	2148.3	1058.8	9706.9	12.8	74.5
**ARZ 5**	416.6	2110.8	1005.2	9938.8	7.2	75.5
**ARZ 6**	601.4	1479.9	900.3	6764.6	16.1	94.8
**COR 1**	541.0	1757.1	421.7	6331.1	22.0	46.2
**COR 2**	1339.6	2843.1	812.8	7610.3	26.6	40.7
**COR 3**	874.9	1716.9	736.4	6320.4	28.4	78.4
**COR 4**	645.9	1079.3	330.7	6145.3	10.3	48.0
**COR 5**	874.5	1887.6	638.3	4341.4	10.8	69.4
**HOJ 1**	-	1060.9	232.8	5406.4	18.8	37.1
**HOJ 2**	1288.6	2830.6	1083.4	8088.1	28.2	71.4
**HOJ 3**	455.3	1494.9	741.3	6646.7	9.4	72.1
**HOJ 4**	214.4	1128.5	290.3	6530.6	7.8	26.5
**HOJ 5**	561.6	1287.0	647.3	7152.2	23.9	78.8
**HOJ 6**	648.0	1382.4	587.2	8418.2	18.8	56.0
**HOJ 7**	837.2	1568.4	412.3	9262.6	10.9	76.0
**PIC 1**	474.7	1137.9	543.9	5883.2	18.3	128.3
**PIC 2**	526.4	1626.8	603.5	6377.3	11.6	91.4
**PIC 3**	803.1	2226.3	715.4	6062.6	-	86.0
**PIC 4**	64.4	1206.8	387.3	7418.1	13.9	97.3
**PIC 5**	1227.2	2718.1	805.9	7510.8	13.2	105.5
**PIC 6**	877.7	2045.2	646.4	8378.2	38.4	-
**PIC 7**	642.0	2111.0	813.8	7120.7	13.6	77.6

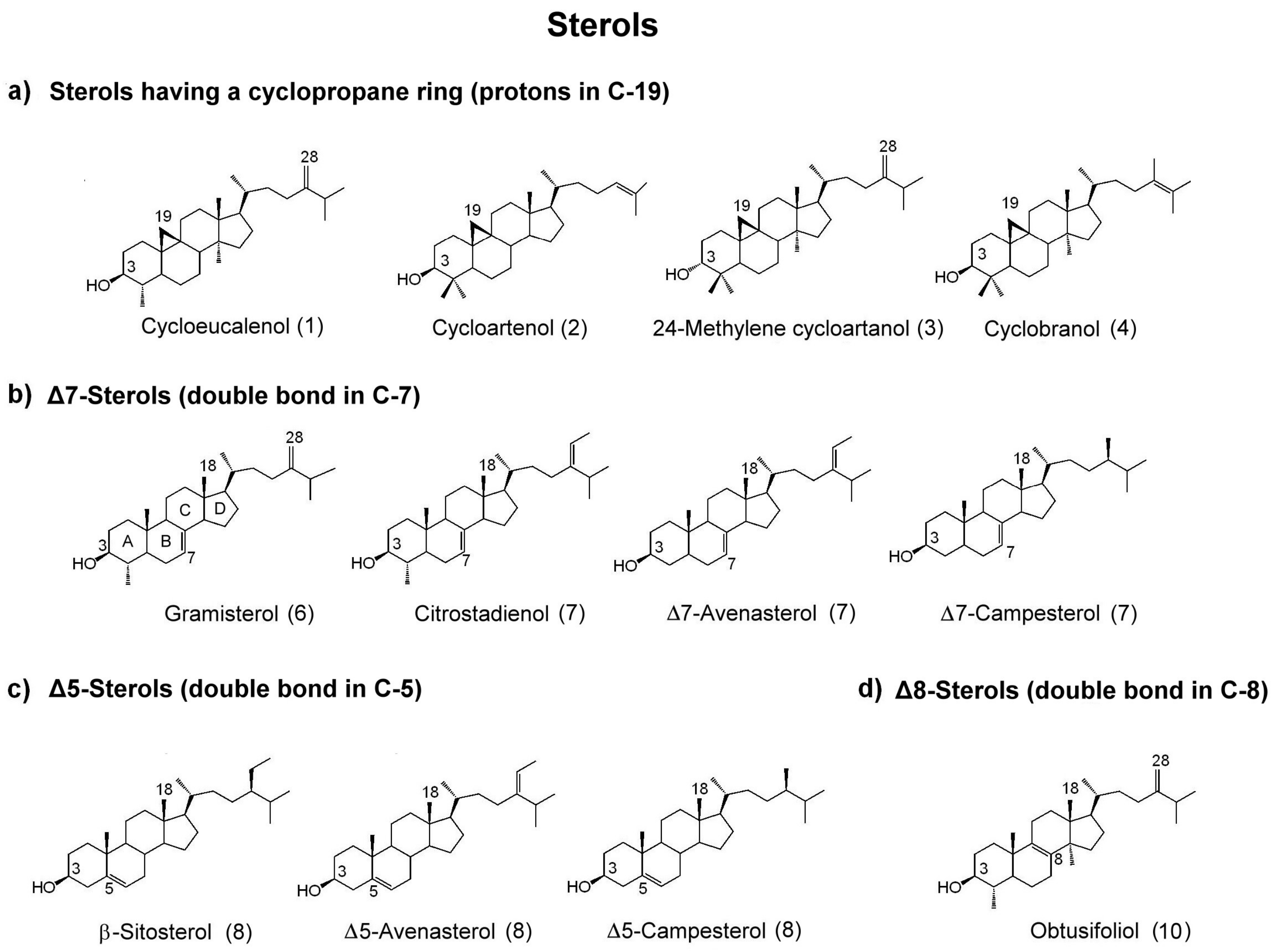

Figure S1. Chemical structures of olive oil sterols: **a**) Sterols having a cyclopropane ring (protons in C-19), such as cycloartenol, 24-methylenecycloartanol, cyclobranol and cycloeucalenol; **b**) Δ7-Sterols (double bond in C-7), such as gramisterol, citrostadienol, Δ7-avenasterol and Δ7-campesterol; **c**) Δ5-Sterols (double bond in C-5), such as β-sitosterol, Δ5-avenasterol and Δ5-campesterol; **d**) Δ8-Sterols (double bond in C-8), such as obtusifoliol. The carbon atoms are numbered in agreement with Table 1.

a) Terpenoids

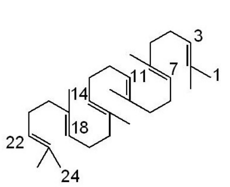

Squalene (9)

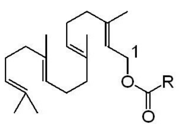

Geranylgeraniol ester

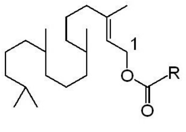

Phytol ester

Non-cyclic diterpenic esters (wax esters,11)

b) Phenolic and secoiridoid derivatives

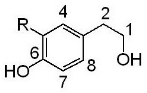

R = OH: Hydroxytyrosol (19)
R = H: Tyrosol (21)

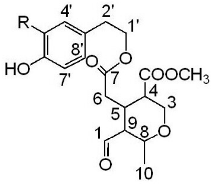

R = OH: 3,4-DHPEA-EA (32)
R = H: *p*-HPEA-EA (31)

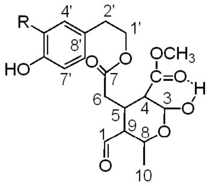

R = OH: Oleomissional (34)
R = H: Oleokoronal (35)

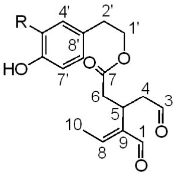

R = OH: 3,4-DHPEA-EDA (oleacein) (26)
R = H: *p*-HPEA-EDA (oleocanthal) (29)

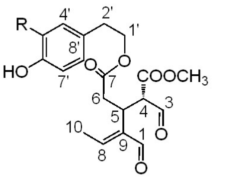

R = OH: 5S,4R-Oleuropeindial (24)
R = H: 5S,4R-Ligstrodial (27)

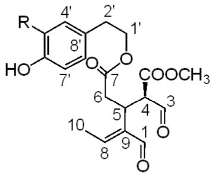

R = OH: 5S,4S-Oleuropeindial (25)
R = H: 5S,4S-Ligstrodial (28)

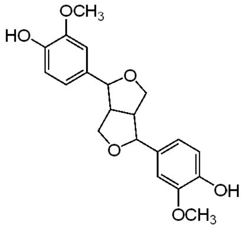

Pinoresinol (22)

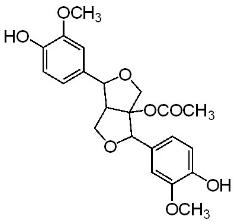

1-Acetoxypinoresinol (23)

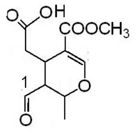

Elenolic acid (33)

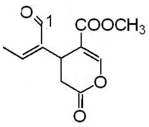

Elenolide (30)

Figure S2. Chemical structures of **a**) Terpenoids, such as squalene and non-cyclic diterpenyl esters, like geranylgeraniol and phytol esters; **b**) Phenolic and secoiridoid derivatives. The carbon atoms are numbered in agreement with Table 1.

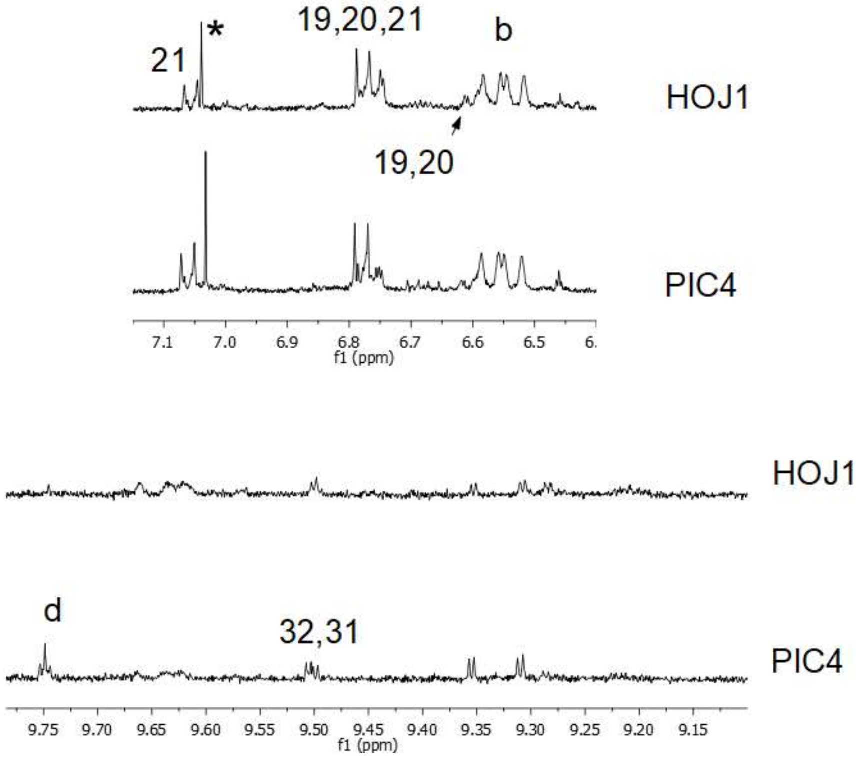

Figure S3. Two expanded regions of the ^{1}H NMR spectra of EVOO samples HOJ1 and PIC4, obtained using the multisuppression experiment (MS). Signal letters and numbers agree with those in Table 1. * Asterisked peak is a side band of chloroform.

^{1}H NMR spectra of standards of some EVOO components spiked in oil (signal number or letter given in brackets):

- Cycloartenol (2)
- β-sitosterol (8)
- Δ5-campesterol (8)
- Δ5-avenasterol (8)
- Squalene (9)
- Hydroxytyrosol (19)
- Tyrosol (21)
- Pinoresinol (22)
- 1,2-dioleoylglycerol (a)
- E-2-hexenal (c)
- Hexanal (d)

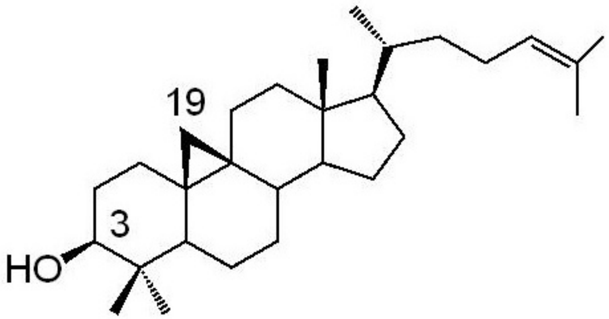

Cycloartenol (2)

spiked in oil

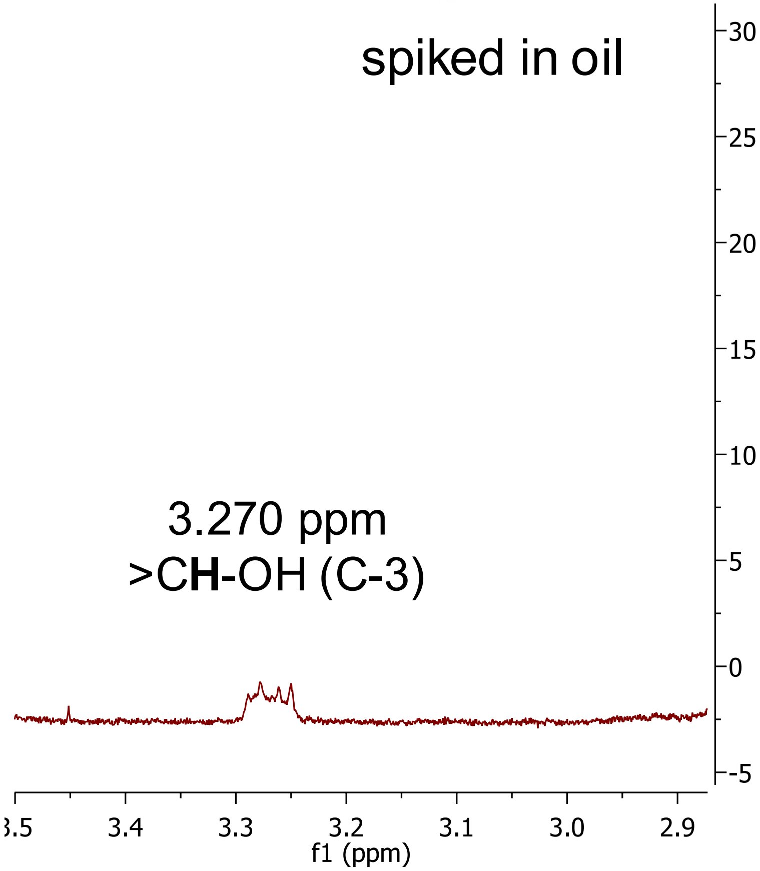

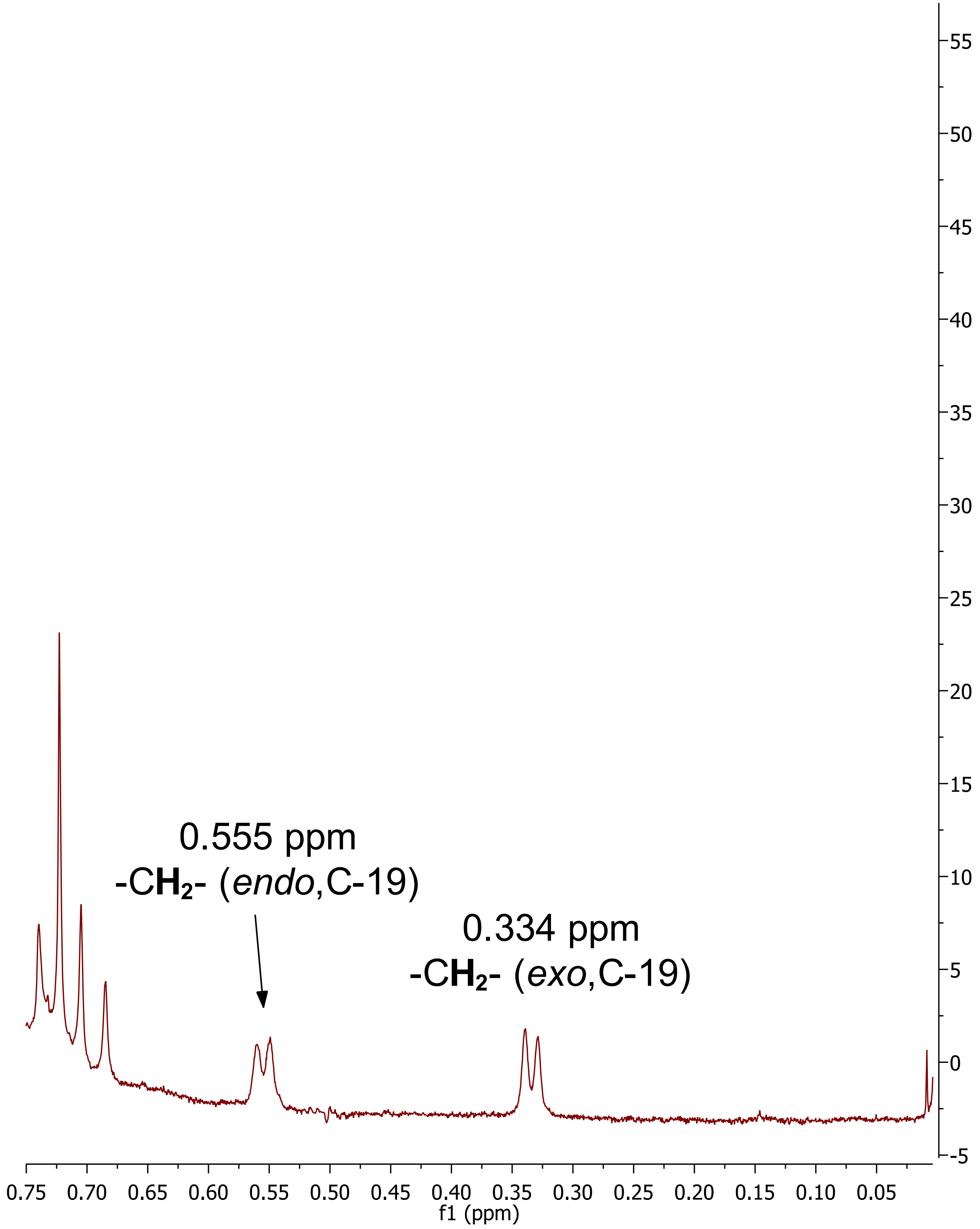

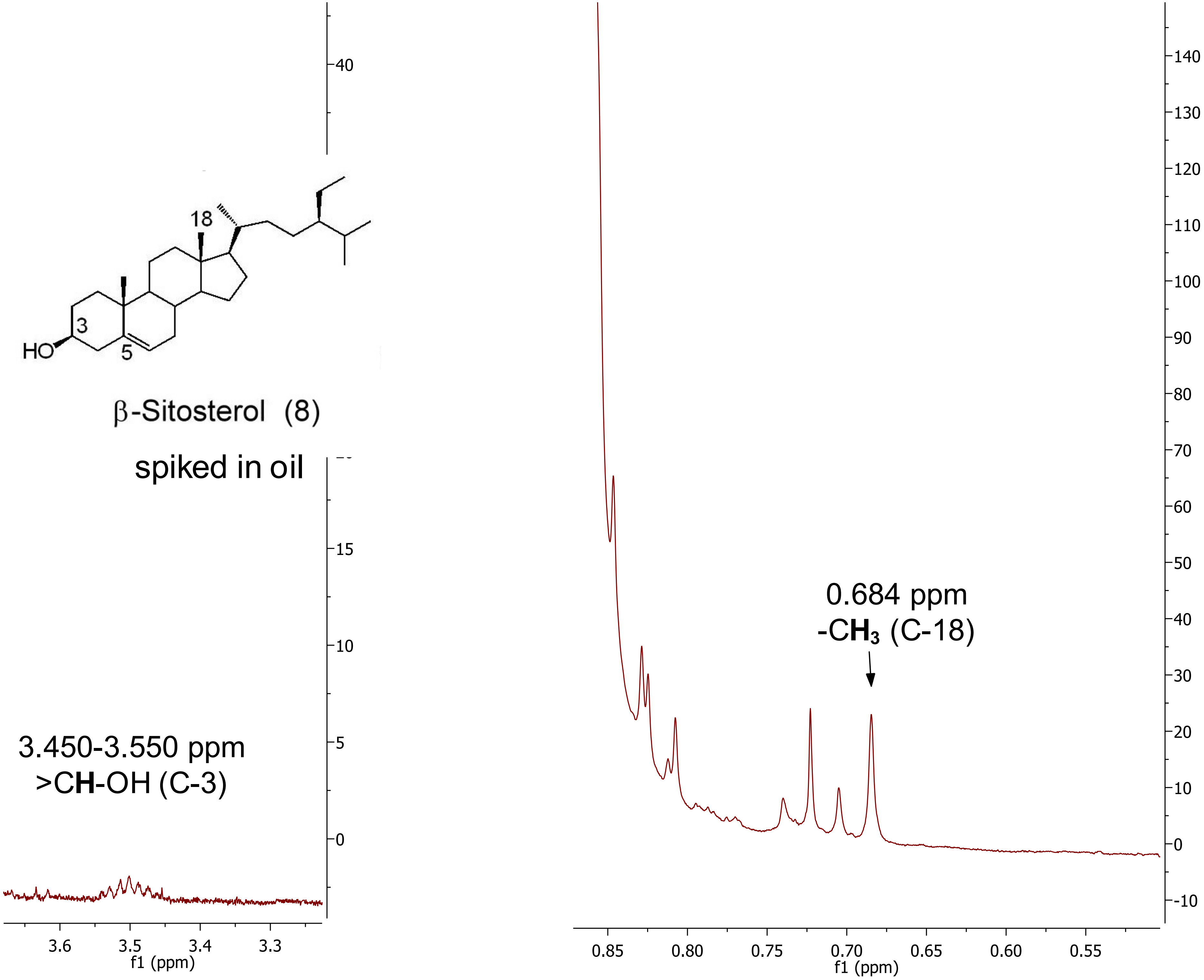

18
3
HO
5
β-Sitosterol (8)
spiked in oil
3.450-3.550 ppm
>CH-OH (C-3)
0.684 ppm
-CH3 (C-18)
f1 (ppm)
f1 (ppm)

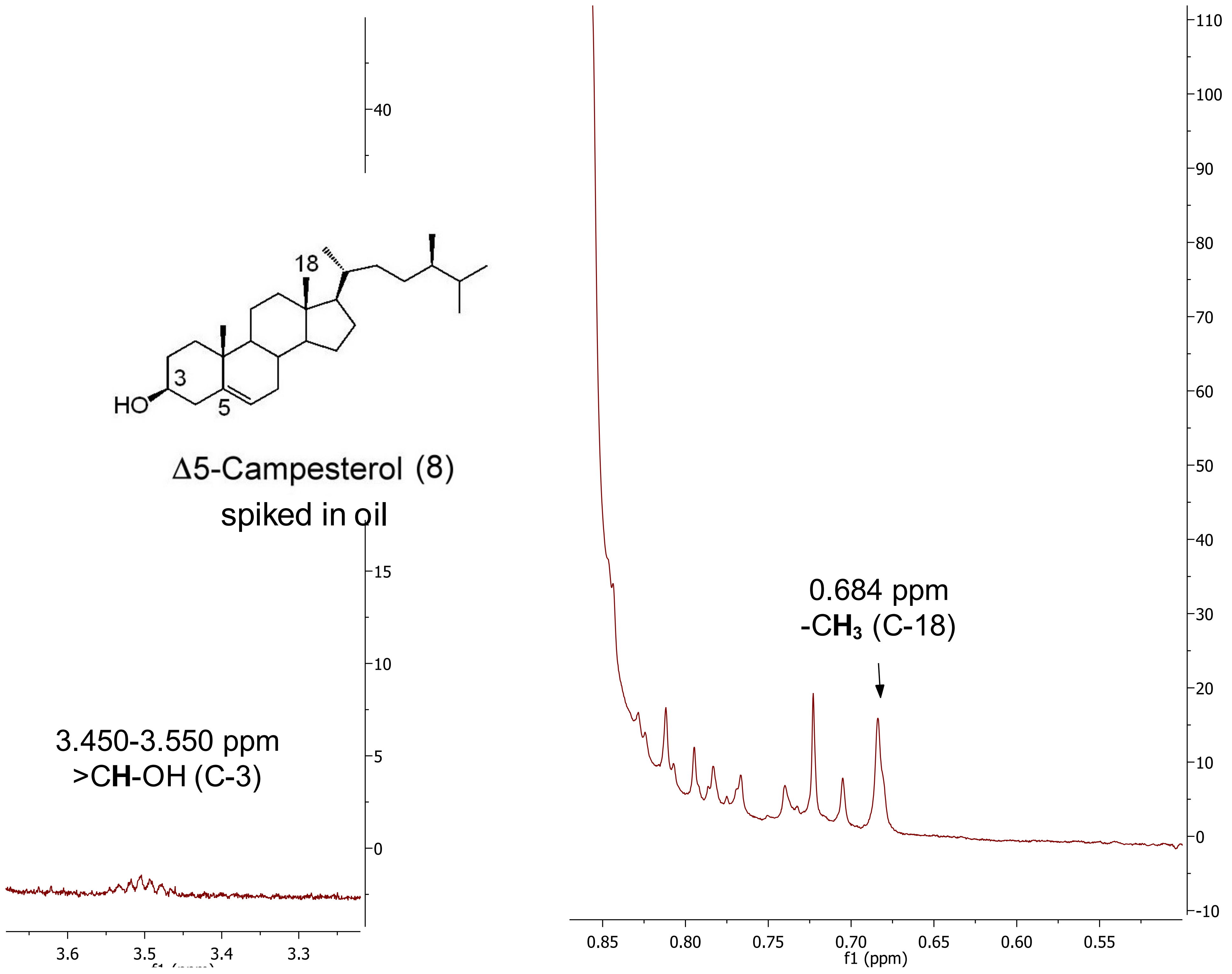

18
3
5
HO
Δ5-Campesterol (8)
spiked in oil
3.450-3.550 ppm
>CH-OH (C-3)
0.684 ppm
-CH3 (C-18)
f1 (ppm)

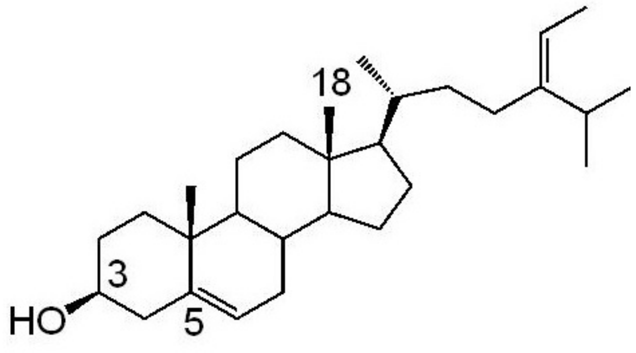

Δ5-Avenasterol (8)

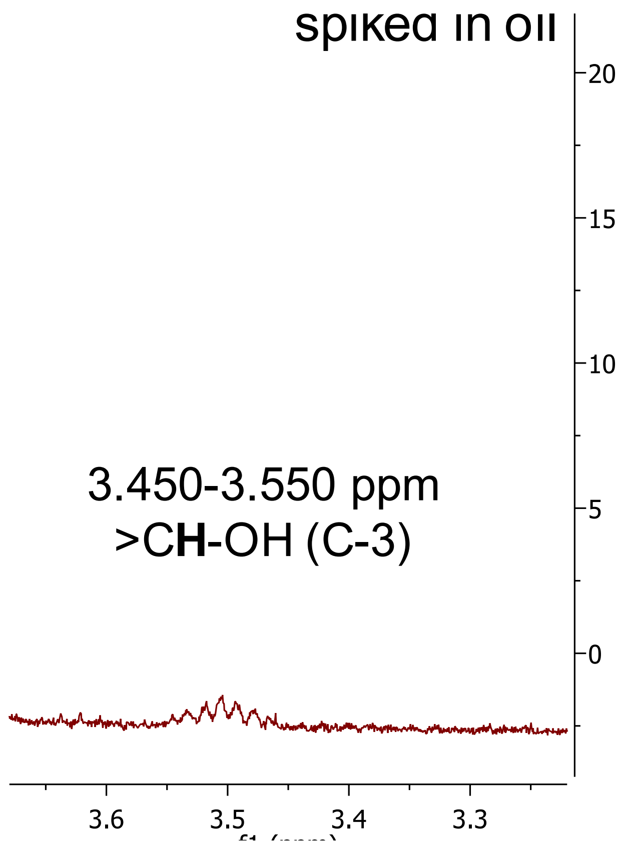

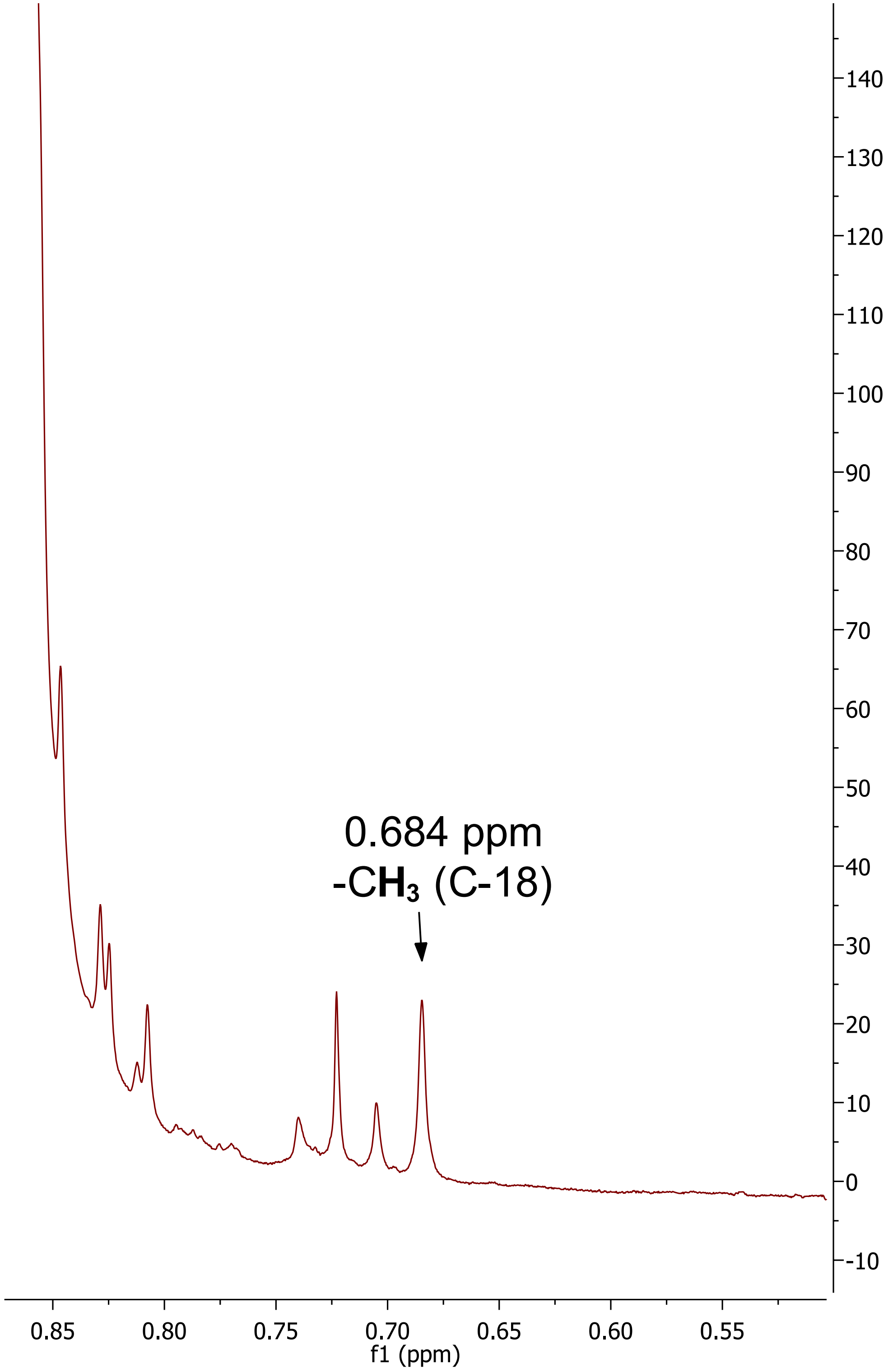

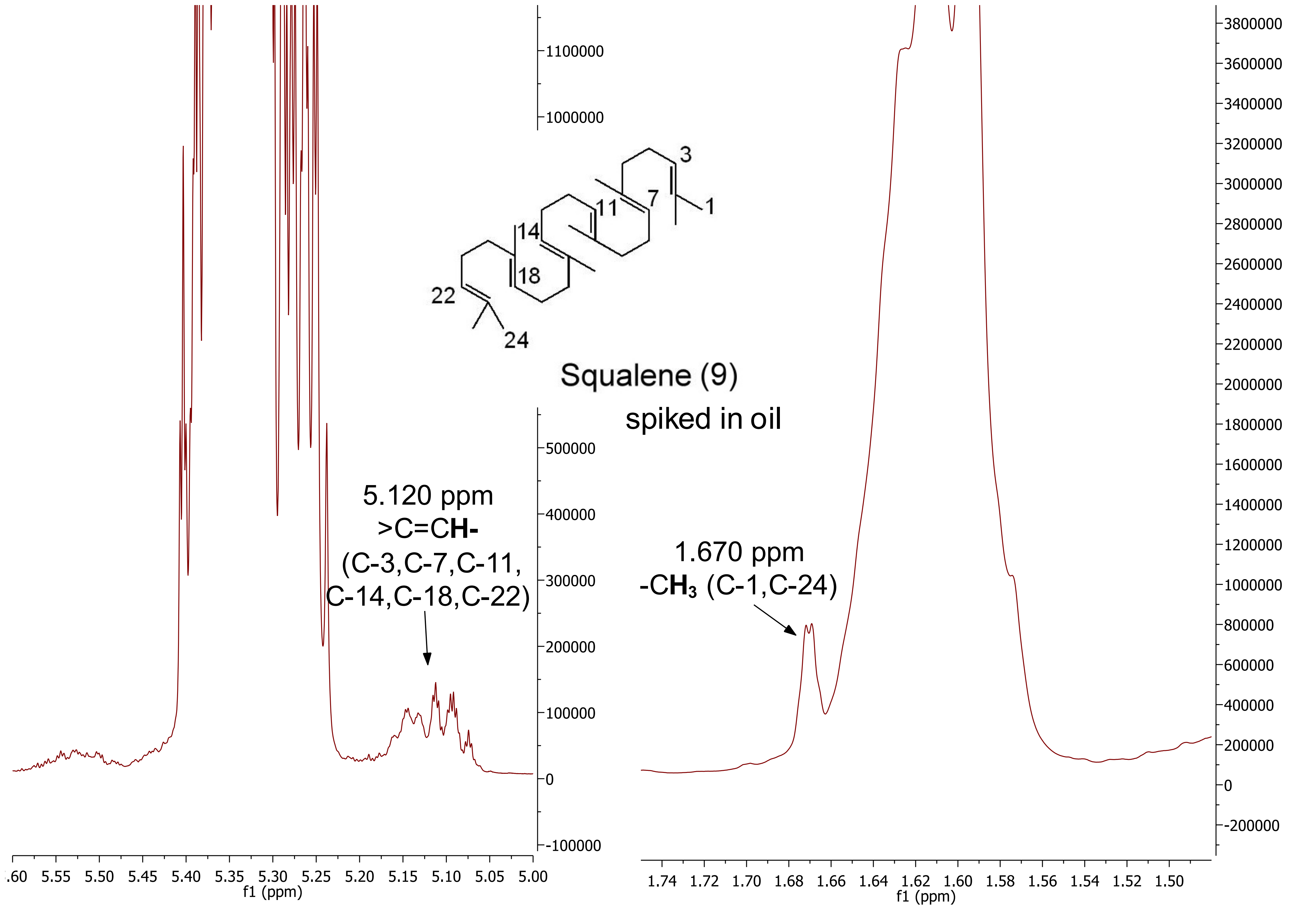

Squalene (9)
spiked in oil
3
1
7
11
14
18
22
24
5.120 ppm
>C=CH-
(C-3,C-7,C-11,
C-14,C-18,C-22)
1.670 ppm
-CH3 (C-1,C-24)
1100000
1000000
500000
400000
300000
200000
100000
0
-100000
.60 5.55 5.50 5.45 5.40 5.35 5.30 5.25 5.20 5.15 5.10 5.05 5.00
f1 (ppm)
3800000
3600000
3400000
3200000
3000000
2800000
2600000
2400000
2200000
2000000
1800000
1600000
1400000
1200000
1000000
800000
600000
400000
200000
0
-200000
1.74 1.72 1.70 1.68 1.66 1.64 1.62 1.60 1.58 1.56 1.54 1.52 1.50
f1 (ppm)

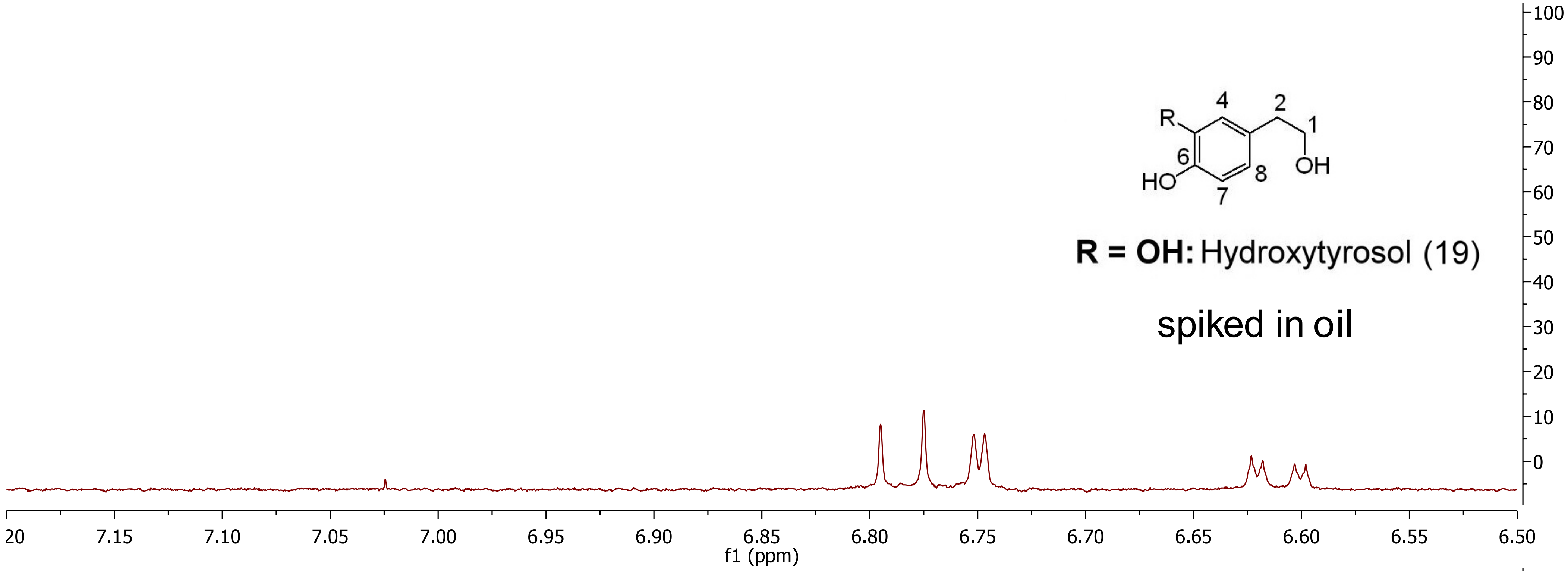

4
2
1
R
6
8
OH
HO
7
R = OH: Hydroxytyrosol (19)
spiked in oil
100
90
80
70
60
50
40
30
20
10
0
20
7.15
7.10
7.05
7.00
6.95
6.90
6.85
6.80
6.75
6.70
6.65
6.60
6.55
6.50
f1 (ppm)

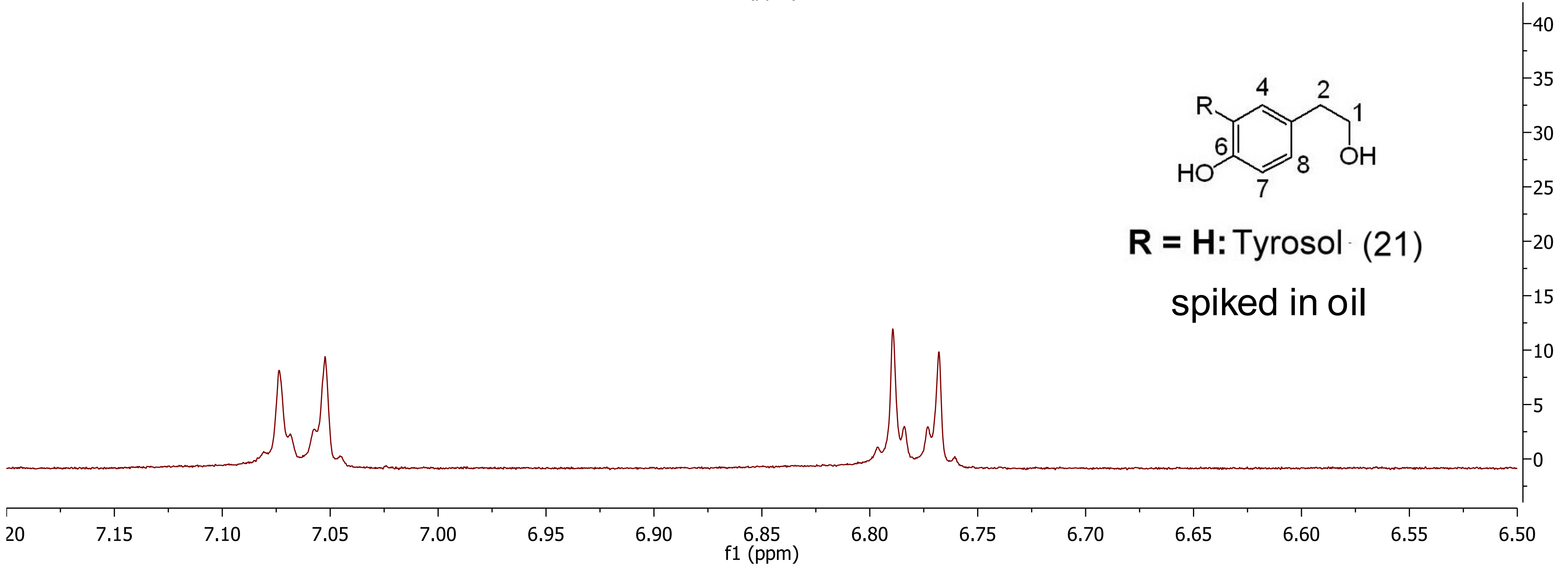

4
2
1
R
6
8
OH
HO
7
R = H: Tyrosol (21)
spiked in oil
40
35
30
25
20
15
10
5
0
20
7.15
7.10
7.05
7.00
6.95
6.90
6.85
6.80
6.75
6.70
6.65
6.60
6.55
6.50
f1 (ppm)

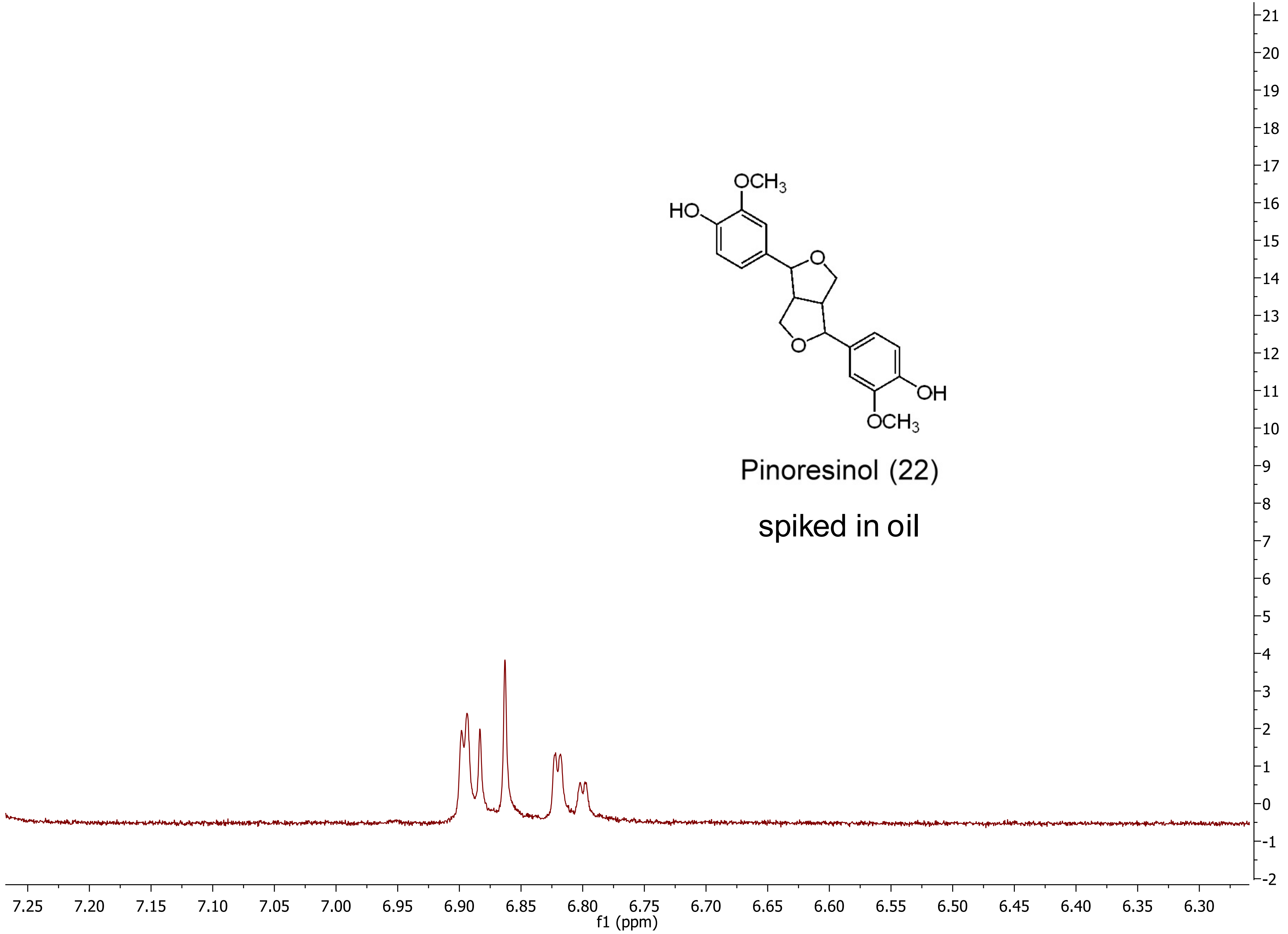

OCH3
HO
O
O
OH
OCH3
Pinoresinol (22)
spiked in oil
21
20
19
18
17
16
15
14
13
12
11
10
9
8
7
6
5
4
3
2
1
0
-1
-2
7.25
7.20
7.15
7.10
7.05
7.00
6.95
6.90
6.85
6.80
6.75
6.70
6.65
6.60
6.55
6.50
6.45
6.40
6.35
6.30
f1 (ppm)

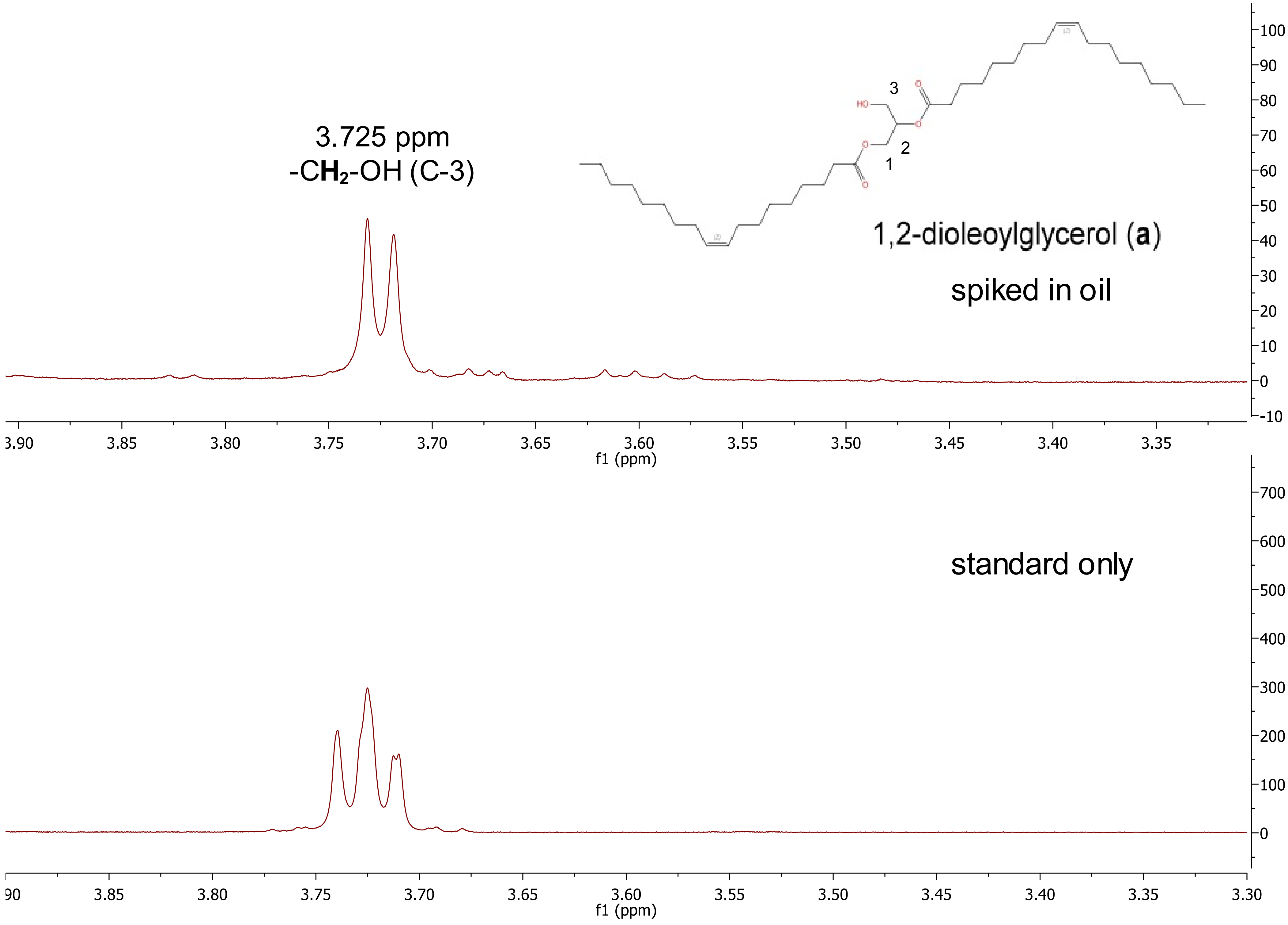
3.725 ppm
-CH$_2$-OH (C-3)
1,2-dioleoylglycerol (**a**)
spiked in oil
standard only
HO
3
2
1
f1 (ppm)
f1 (ppm)

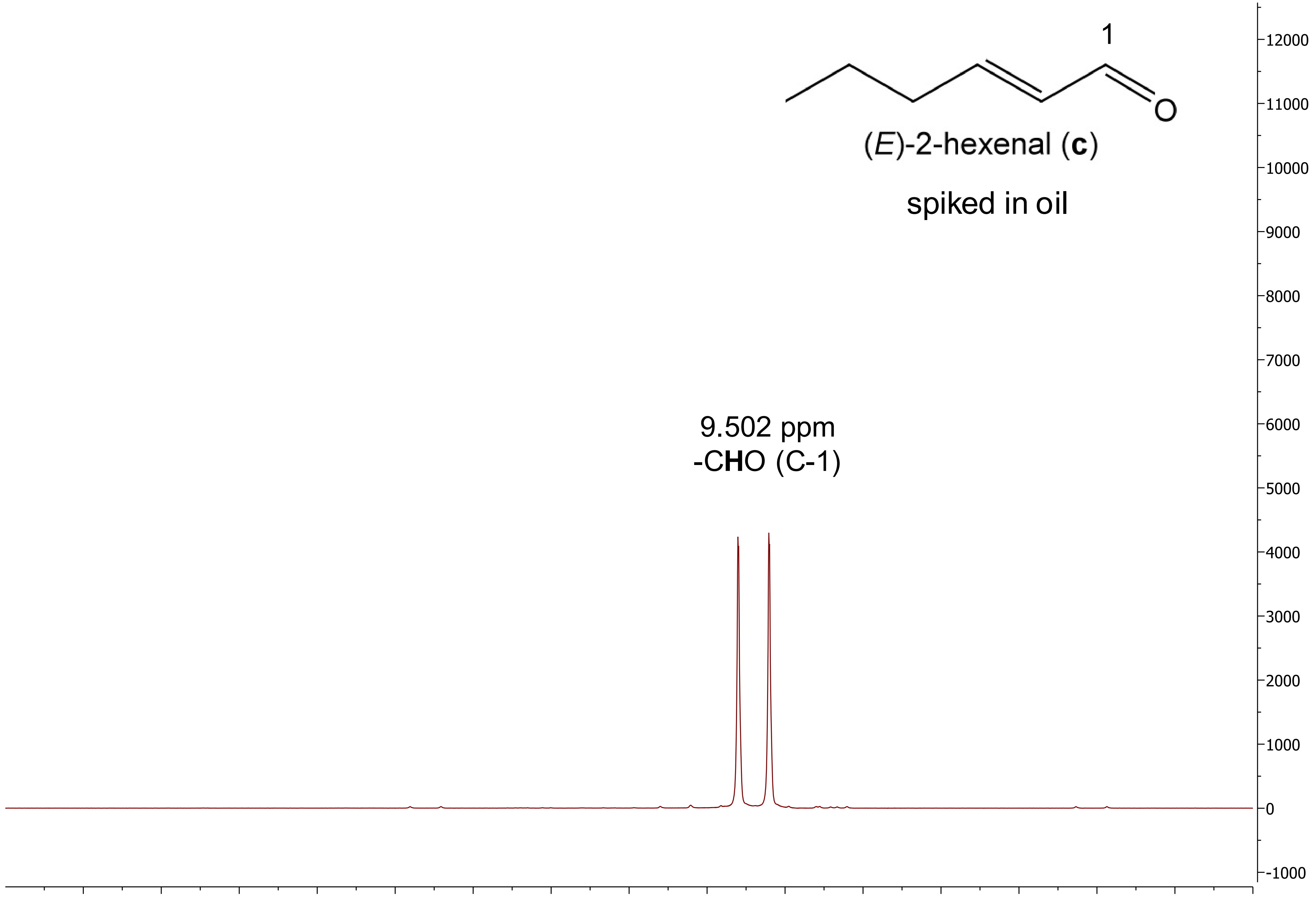
1
O
(E)-2-hexenal (c)
spiked in oil
9.502 ppm
-CHO (C-1)
12000
11000
10000
9000
8000
7000
6000
5000
4000
3000
2000
1000
0
-1000

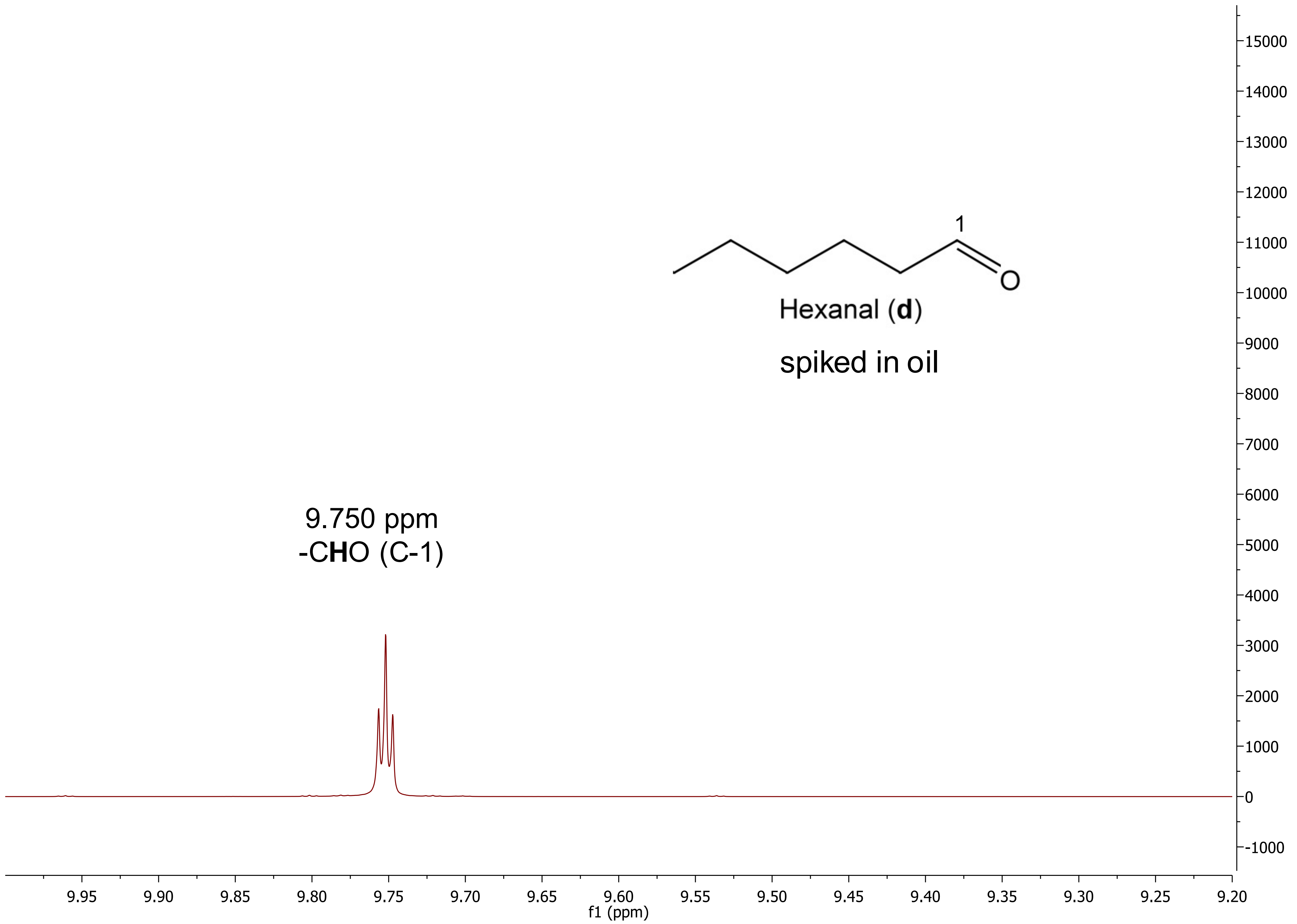
1
O
Hexanal (d)
spiked in oil
9.750 ppm
-CHO (C-1)
15000
14000
13000
12000
11000
10000
9000
8000
7000
6000
5000
4000
3000
2000
1000
0
-1000
9.95
9.90
9.85
9.80
9.75
9.70
9.65
9.60
9.55
9.50
9.45
9.40
9.35
9.30
9.25
9.20
f1 (ppm)
